# Quantifying the exposure-response relationship between temperature exposure and semen quality

**DOI:** 10.3389/fpubh.2026.1813888

**Published:** 2026-04-13

**Authors:** Guangyuan Liu, Jiaxin Liu, Yawen Liu, Shuren Sun, Qi Chen, Hong Huang, Zhigang Wu

**Affiliations:** 1 School of Public Health, Wenzhou Medical University, Wenzhou, China; 2 Department of Infection Prevention and Control and Preventive Medicine, Zhejiang Hospital, Hangzhou, China; 3 Ouhai District Center for Disease Control and Prevention of Wenzhou, Wenzhou, China; 4 Department of Urology, The First Affiliated Hospital of Wenzhou Medical University, Wenzhou, China

**Keywords:** excessive temperature, exposure-response, nonlinear, optimal temperature, threshold, semen quality

## Abstract

**Background:**

High temperature exposure impacts semen quality, but the exposure-response relationship needs further study.

**Objectives:**

Quantify the correlations and nonlinear relationships between apparent temperature (AT) exposure and semen quality parameters.

**Methods:**

A total of 5,114 individuals were enlisted as participants from a reproductive medicine center in Wenzhou, a developed coastal cities in eastern China. Multivariate linear regression models were used to quantify the correlations between AT exposure and semen quality parameters. Restricted cubic spline (RCS) and piecewise regression were employed to validate the nonlinear and threshold effects of the exposure-response relationships.

**Results:**

Each 1 °C rise in AT corresponded to considerable declines in progressive motility (−2.1165; 95% CI: −2.6219, −1.6111) and total motility (−4.8823; 95% CI: −6.2494, −3.5151). AT negatively influenced sperm motility during lag periods of 15–69 and 70–90 days (*p* < 0.05). Nonlinear RCS analysis detected an inverse U-shaped association between AT and sperm motility, especially at lags 10–14 days (nonlinear *p* < 0.05). The AT range associated with optimal semen quality parameters was approximately 17.57–22.69 °C based on segmented analysis.

**Conclusions:**

Elevated exposure to apparent temperature in the 90 days prior to donation was associated with reduced semen quality. Avoiding excessive temperature exposure, particularly within a 2-week pre-ejaculation window, helps to maintain ideal semen quality.

## 1 Introduction

Infertility is a significant issue impacting regional politics and economy. Approximately 8%−12% of couples worldwide encounter this issue, and notably, male-factor infertility contributes to causative factors in 50% of such instances (1). A comprehensive diagnostic approach in a large cohort of 1,014 couples with primary infertility revealed that isolated male factor was present in 23% of couples, while in 45% it was associated with concurrent female factors (2). Semen quality parameters are a critical clinical indicator that can significantly impact male fertility (3). However, decreases in semen quality parameters among males have been documented in numerous studies (4–6).

Many factors can affect semen quality, including genetic factors, lifestyle, and work environment. Nowadays, people have generally recognized that environmental pollution and climate change are important reasons for the decline in semen quality. Recent decades have seen climate change, characterized by surging temperatures, has unleashed a cascade of interconnected ecological crises. Collectively, these environmental changes—particularly the rise in ambient temperatures—pose an emerging threat to male reproductive health (7). The detrimental effects of extreme temperatures on semen quality have been well-documented in various animal models, including murine, bovine, canine, and lapine species, providing robust evidence for the thermal sensitivity of male reproductive function (8–14). Presently, the available evidence is insufficient to decisively confirm the relationship between temperature and semen quality, as findings from different studies show inconsistencies (15–19). Zhou et al. (18) observed that sperm concentration, total count, and motility decreased significantly when average temperatures deviated from 13 °C during the 0–90 days prior to collection in Wuhan, China. Similarly, a retrospective cohort study in Guangdong (15) reported that temperature exposure during the same window was associated with reduced sperm counts. In contrast, Momen et al. (20) conducted a prospective study and found that semen parameters remained within clinically normal ranges despite elevated ambient temperatures. These inconsistencies may reflect differences in study design, population susceptibility, or climate characteristics underscoring the need for further research to clarify this relationship. Given that humans concurrently experience a multitude of atmospheric environmental elements, employing a combined bioclimatic index known as AT (21), which integrates several environmental variables including temperature, relative humidity, and wind speed, serves to more accurately represent how the human body perceives actual temperature (22). In addition, climate change can affect air quality, and air pollutants can in turn boost climate change, thereby affecting public health (23).

Understanding the relationship between temperature exposure and semen quality across different time frames is pivotal for effective public health interventions. The approximate time frame for the full development of human spermatozoa is 90 days (24). Prior research has identified key stages of sperm development—spermatogenesis (70–90 days pre-collection), motility development (10–14 days pre-collection), and epididymal storage (0–9 days pre-collection)—that may be influenced by temperature variations (17, 25–28). Regrettably, the number of studies examining the correlation between temperature and semen quality across varying exposure durations is quite limited (15, 17, 29). Recently, some studies have defined the period of 15–69 days before semen sampling as “spermatogenesis stage II” (30, 31). Although this classification is not absolutely precise, it is grounded in the temporal logic of spermatogenesis. This approach facilitates a focused assessment of the sensitivity of this biological stage to temperature while controlling for potential confounding from non-critical developmental phases. Based on this, the present study incorporated the 15–69 days window as an independent stage in the model to more accurately capture the effect of the critical exposure window. The influence of temperature on semen quality exhibits regional disparities, necessitating a thorough examination of the temperature-seminal quality relationship across diverse geographic contexts. Taking Wenzhou as the study area, this paper tried to estimate the effects of AT exposure on the semen quality and additionally identify periods of heightened influence. Major air pollutants (PM_2.5_, PM_10_, SO_2_, NO_2_, O_3_, and CO) are regarded as covariates, and principal component analysis (PCA) is utilized to reduce the impact of multicollinearity (32). Furthermore, we conducted stratified analyzes to determine whether this correlation varied with age or season.

## 2 Method

### 2.1 Study area and population

Wenzhou's urban districts, encompassing Lucheng District, Longwan District, and Ouhai District, are situated within China's eastern coastal belt, covering a land expanse of 1,075 km^2^. Characterized by a subtropical monsoon climate, these areas experience an annual average temperature of 17.3–20.0 °C and an annual precipitation of 1,023–2,494 mm. With a permanent resident population of 2,959,100, the urban areas boast a per capita Gross Domestic Product (GDP) of 309,946 yuan, classifying it as an economically developed region.

**Figure 1:**
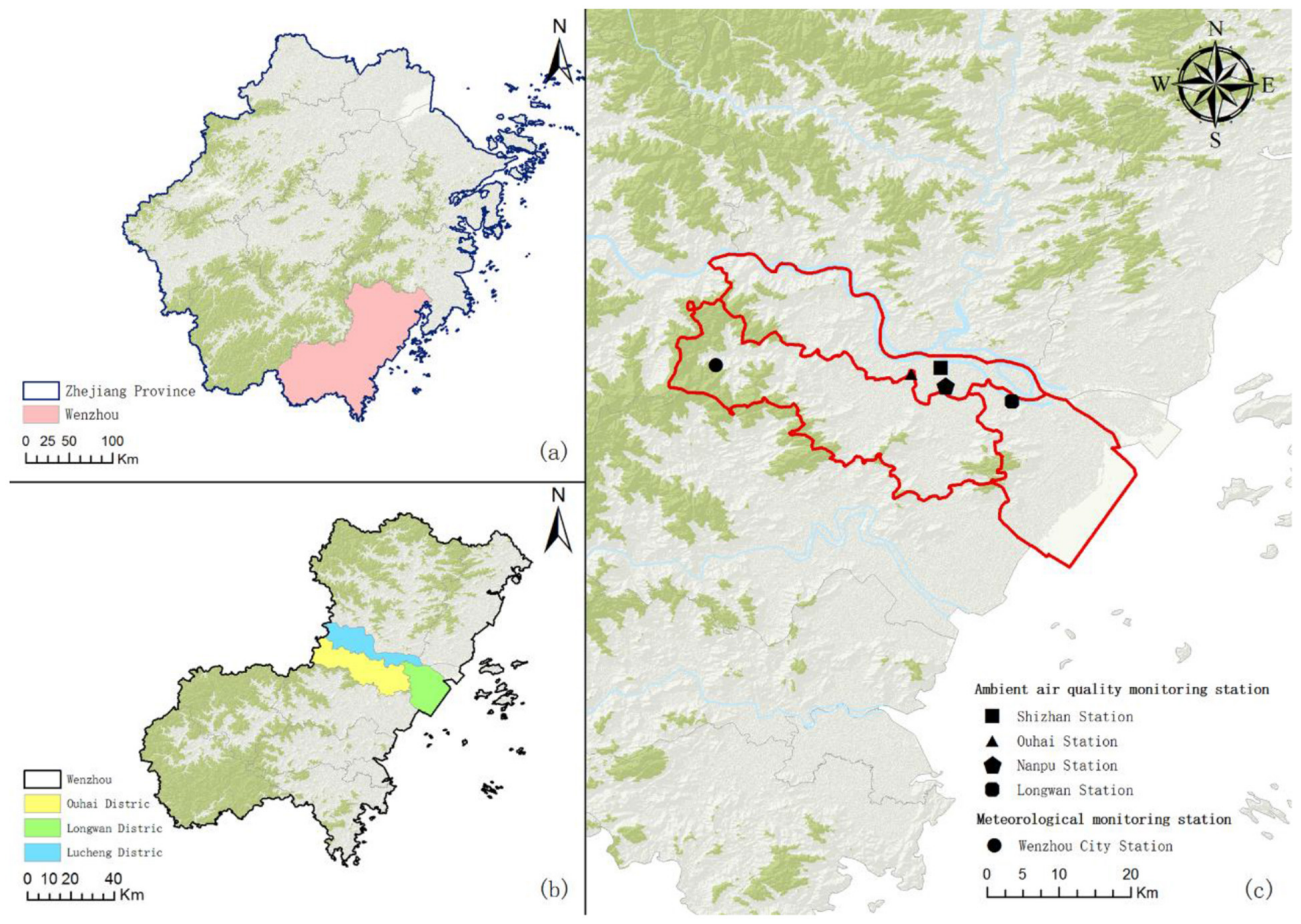
Spatial distribution of the study area (*n* = 3) and ambient air quality monitoring station (*n* = 4). **(a)** Location of Wenzhou City in Zhejiang Province; **(b)** Location of Wenzhou urban districts (Lucheng District, Ouhai District, Longwan District); **(c)** Spatial distribution of ambient air quality monitoring stations and meteorological monitoring station in Wenzhou City.

Participants were recruited from the Reproductive Medicine Center of a hospital in Wenzhou, China. Between May 2014 and December 2022, a total of 63,680 men underwent semen analysis at this institution for either fertility evaluation or routine health checks. We restricted our investigation to individuals residing in the primary urban areas of Wenzhou (Figure 1) to ensure spatial consistency with exposure monitoring sites. The following exclusion criteria were applied: (1) residence outside the Wenzhou urban area; (2) semen testing date outside the study period; (3) missing demographic or clinical records; (4) repeated samples from the same individual (only the first sample was retained); and (5) presence of documented reproductive disorders that could affect semen quality (e.g., epididymitis, mumps, asymptomatic andrological conditions including chlamydial infections, Klinefelter syndrome, varicocele, orchiectomy or seminal vesiculectomy, absence of the vas deferens, and Y chromosome microdeletion), based on criteria established in previous studies (33, 34). Following application of these criteria, the final analytical sample comprised 5,114 participants. Supplementary Figure S1 provides a detailed overview of the screening process.

### 2.2 Semen analysis

After a period of abstinence ranging from 2 to 7 days, participants were only able to be evaluated by donating a sample of their sperm by masturbating in the collecting room. A computer-assisted sperm analysis (CASA) system (MICROPTIC S. L. Company, Viladomat, Barcelona, Spain) was utilized in order to identify and evaluate the various factors that pertain to the quality of semen (35). The five indicators of semen quality that were taken into consideration were progressive motility (%), total motility (%), sperm concentration (10^6^/ml), total sperm number (10^6^) and semen volume (ml). These parameters were evaluated in strict adherence to the stipulations outlined by the World Health Organization's (WHO) established guidelines (36).

### 2.3 Environment exposure assessment

The meteorological data including atmospheric pressure (hPa), ambient temperature (°C), precipitation (mm), sunshine duration (h), relative humidity (%) and wind speed (m/s) utilized in this study were acquired from the National Meteorological Science Data Center (http://data.cma.cn/), while the monitoring data of air pollutants including PM_2.5_ (μg/m^3^), PM_10_ (μg/m^3^), SO_2_ (μg/m^3^), NO_2_ (μg/m^3^), O_3_ (μg/m^3^), and CO (mg/m^3^) were collected from the National Urban Air Quality Real-time Release Platform (https://air.cnemc.cn:18007/). Due to privacy protection measures, precise residential addresses of participants were unavailable, precluding individual-level exposure assessment. We therefore used daily averages from four urban air quality monitoring stations and the Wenzhou meteorological station as indicative measures of participants' daily exposure levels. Given the close spatial proximity of these monitoring sites (Figure 1), the use of city-wide averages is likely to provide a robust proxy for background ambient exposure, thereby minimizing the potential for large-scale spatial exposure contrasts among the study participants (33). we adopt daily average data from four pollution monitoring sites and daily data from the Wenzhou weather station as indicative measures of participants' daily exposure levels. All data were expressed as 24-h averages, and missing data were imputed using the univariate ARIMA state space model and Kalman smoothing (37). The AT was determined by applying the following (Equations 1, 2) (38):


$$\begin{array}{l} AT=T_{a}+0.33\;*\;e-0.70\;*\;WS-4.00 \end{array}$$ (1)

$$\begin{array}{l} e=\frac{Rh}{100}\;*\;6.105\;*\;exp\left(\frac{17.27*T_{a}}{237.7+T_{a}}\right) \end{array}$$ (2)

where *T*_*a*_ represents the ambient temperature (°C), *WS* represents wind speed (m/s), *Rh* represents the relative humidity (%), *e* represents the water vapor pressure which is calculated using Equation 2.

To quantify the individual exposure level of each participant, we computed the mean value of each environmental component variable across five exposure windows (0–90, 0–9, 10–14, 15–69 and 70–90 days before semen examination).

### 2.4 Statistical analysis

We utilized percentiles or median (lower quartile, upper quartile) to characterize all variables that did not follow a normal distribution. Additionally, we conducted a Box-Cox transformation (Supplementary Figures S1.1–S1.4) for all quality parameters. The general form of Box-Cox transformation is as follows (Equation 3):


$$y^{\left(\lambda \right)}=\{\begin{array}{c} \frac{{\left(y+a\right)}^{\lambda }-1}{\lambda },\lambda \neq 0\\ In(y+a),\lambda =0 \end{array}$$ (3)

Where *y* is the original continuous dependent variable (progressive motility, total motility, total sperm number, sperm concentration and semen volume), *y*^(λ)^ is the new variable obtained after the Box–Cox transformation, and λ is the transformation parameter. The above transformation requires that the variable *y* take on a positive value. If the value of the formula (*y*+*a*) positive, and then the above transform is performed. By consulting the literature, we determined that co-predictors of apparent temperature exposure and semen quality could serve as potential confounders, in accordance with the disjunctive cause criterion (39). Utilizing DAGitty v3.1 software (Johannes Textor Institutional Affiliation (v3.1 era), Data Science group, Radboud University, Nijmegen, Netherlands) (http://www.dagitty.net), we created a directed acyclic graph (DAG) to illustrate their interrelationships visually (40) (Supplementary Figure S2). Spearman correlation analysis was used to identify factors that require control in order to mitigate the bias of association estimates. Specifically, there were notable correlations between some air pollutants (such as PM_10_ and NO_2_, *r* = 0.71, Supplementary Figure S3), we performed principal component analysis to convert air pollutants, and incorporated principal component 1, which exhibited the highest variance, as a confounding factor in the analysis to mitigate collinearity. Ultimately, the following covariates were incorporated into the analysis: age (30, 31–39, 40 years, unknown), ever having fathered a child (yes, no), smoking (yes, no), alcohol consumption (yes, no), education (college and higher, high school, middle school and lower, unknown), occupation (worker, businessman, peasant, intellectual, others, unknown), abstinence periods (2–3, 4–5, 6–7 days), season of sperm collection (spring, summer, autumn, winter), average daily precipitation (continuous), average daily sunshine duration (continuous), and the first principal component derived from PCA-transformed average daily air pollutants (including PM_2.5_, PM_10_, SO_2_, NO_2_, O_3_ and CO, continuous).

We used linear regression models to precisely quantify the correlation between exposure to AT and semen quality parameters. In categorical analyses, AT exposure was separated into quantiles, with the first quantile serving as the reference. Linear trend tests were conducted by replacing the original value with the median value for each quintile. Additionally, to explore the potential nonlinear association between AT exposure and semen quality, we employed a restricted cubic spline (RCS) model with four knots positioned at the 5th, 35th, 65th, and 95th percentiles of the exposure distribution, a configuration that optimally balances curve smoothness and model flexibility while minimizing the risk of overfitting (41). In addition, we found some inverted U-shaped curve relationships and used piecewise regression based on the highest point of the curve to confirm the nonlinearity of the exposure response.

We performed stratified analyses based on age and season. In addition, we conducted an interaction analysis between stratification variables and AT using likelihood ratio tests to investigate if these factors modified the relationship between apparent temperature exposure and semen quality. We conducted a sensitivity analysis involving: (1) reassessing the nonlinear relationship between AT exposure and semen quality with RCS knots set to 3 and 5, (2) including only subjects with normal semen quality without abnormal sperm parameters (sperm concentration <15 × 10^6^ /ml), total sperm number <39 × 10^6^, total motility <40 %, progressive motility <32 % or semen volume <1.5 ml, (3) excluding participants recruited post-December 2019 due to the COVID-19 pandemic, and (4) excluding all participants with records of unknown values (36).

## 3 Results

### 3.1 Study participants and characteristics

Table 1 and Supplementary Table S1 present the characteristics of the study population, comprising all male participants (*n* = 5,114), a subgroup with normal semen quality (*n* = 3,660), subgroups that were enrolled prior to the COVID-19 outbreak (*n* = 3,663), and a subgroup that excludes any participants with unknown values (*n* = 4,478). The participants were mostly 31–39 years old (55.4%), worker (73.2%) and with education of middle school and lower (38.9%). Furthermore, the majority of individuals were non-smokers (93.4%) and non-drinkers (99.0%), and over half of individuals had never fathered a child (62.0%). Most participants abstained for 4–5 days (50.2%) before semen collection. The semen quality parameters of the subgroups were superior to those of the total participants.

**Table 1:** Demographic characteristics and semen quality of all participants.

Characteristics	All participants (*N* = 5,114)
Age, years, *n* (%)	
≤ 30	1,617 (31.6)
31–39	2,831 (55.4)
≥40	655 (12.8)
Unknown	11 (0.2)
Ever having fathered a child, *n* (%)	
Yes	1,945 (38.0)
No	3,169 (62.0)
Alcohol consumption, *n* (%)	
Yes	52 (1.0)
No	5,062 (99.0)
Smoking, *n* (%)	
Yes	335 (6.6)
No	4,779 (93.4)
Occupation, *n* (%)	
Worker	3,742 (73.2)
Businessman	486 (9.5)
Peasant	41 (0.8)
Intellectual	327 (6.4)
Others	404 (7.9)
Unknown	114 (2.2)
Education, *n* (%)	
College and higher	1,791 (35.0)
High school	747 (14.6)
Middle school and lower	1,988 (38.9)
Unknown	588 (11.5)
Abstinence periods, day, *n* (%)	
2–3	1,635 (32.0)
4–5	2,565 (50.2)
6–7	914 (17.9)
Season, *n* (%)	
Spring (Mar–May)	1,412 (27.6)
Summer (Jun–Aug)	1,399 (27.4)
Autumn (Sep–Nov)	1,277 (25.0)
Winter (Dec–Feb)	1,026 (20.1)
Progressive motility, %	55.30 [40.80, 68.80]
Total motility, %	63.30 [50.10, 76.50]
Sperm concentration, × 10^6^/ml	72.45 [35.90, 122.68]
Total sperm number, × 10^6^	221.75 [108.23, 385.75]
Semen volume, ml	3.20 [2.30, 4.20]

Data were given as number (percent)/median [lower quartile, upper quartile] as indicated.

### 3.2 Environmental exposure

**Figure 2:**
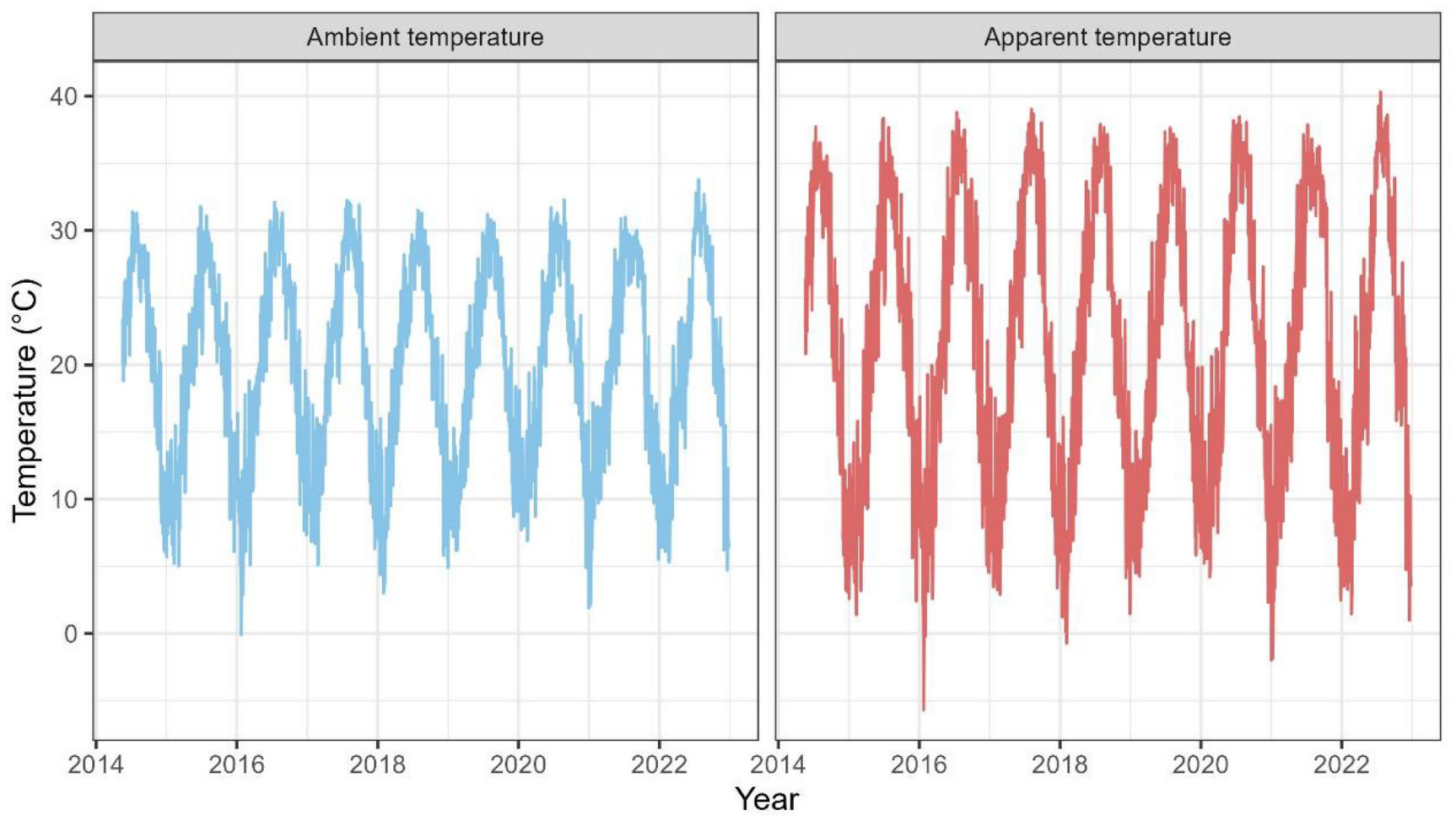
Distributions of ambient temperature and apparent temperature between 2014 and 2022.

Figure 2 and Supplementary Figure S4 present data pertaining to air pollutants and meteorological variables throughout the study duration, manifesting clear seasonal fluctuations. Table 2 and Figure 2 shows that the AT (range: 7.73 °C−35.20 °C) typically exhibits a significant variation between hot and cold, in comparison to the ambient temperature (range: 9.43 °C−29.89 °C). The participants displayed similar distributions of those environmental factors during five exposure periods (Supplementary Table S2). The principal component analysis summarized six air pollutants into six components, with the first component's results selected for further analysis (Supplementary Table S3).

**Table 2:** Summary statistics of meteorological data and air pollutants exposure by lag 0–90 days.

Exposure	Mean ±SD	Minimum	P_25_	Median	P_75_	Maximum
Ambient temperature, °C	19.83 ± 6.28	9.43	13.56	20.21	25.93	29.89
Apparent temperature, °C	21.77 ± 8.74	7.73	13.05	22.10	30.23	35.20
Precipitation, mm	4.89 ± 2.59	0.49	2.69	4.55	6.71	13.30
Sunshine duration, h	3.52 ± 1.06	1.09	2.78	3.36	4.24	6.70
Relative humidity, %	76.99 ± 4.72	64.62	73.63	77.74	80.72	85.21
Wind speed, m/s	0.77 ± 0.08	0.51	0.73	0.77	0.83	0.99
PM_2.5_, μg/m^3^	31.79 ± 9.98	15.37	24.32	30.38	37.60	62.23
PM_10_, μg/m^3^	58.60 ± 14.19	34.11	48.18	56.86	68.89	99.88
SO_2_, μg/m^3^	9.28 ± 4.10	4.24	6.09	7.60	12.00	25.28
NO_2_, μg/m^3^	35.99 ± 8.92	17.66	28.94	35.40	42.27	61.25
O_3_, μg/m^3^	111.30 ± 21.96	55.95	92.48	114.57	129.25	152.58
CO, μg/m^3^	0.69 ± 0.16	0.41	0.55	0.67	0.80	1.13

SD, standard deviation; P_25_, the 25th percentile; P_75_, the 75th percentile.

### 3.3 Exposure-response analysis

Because all semen parameters were Box-Cox transformed to satisfy model assumptions, the reported coefficients represent changes on the transformed scale, not in the original measurement units. Table 3 presents the result of linear regression model of the effect of AT on semen quality parameters during lag 0–90 days. A 1 °C increase of AT was associated with a −2.1165 (−2.6219, −1.6111) and −4.8823 (−6.2494, −3.5151) decrease in progressive motility and total motility (all *p* < 0.001), and this association was also confirmed by liner trend test (*p* < 0.001). Linear trend tests also indicated a potential negative relationship between AT and semen volume (*p* for trend <0.05), although it was not statistically significant (*p* > 0.05). Based on the Box-Cox transformation parameters (λ) detailed in Supplementary Table S1, we back-transformed the regression coefficients to the original scale using the median values of the study population (progressive motility: 55.3%; total motility: 63.3%; semen volume: 3.2 ml). Accordingly, a 1 °C increase in AT corresponds to an approximate decrease of 0.12 percentage points (95% CI: 0.09–0.15) in progressive motility and 0.08 percentage points (95% CI: 0.06–0.10) in total motility.

**Table 3:** Estimated changes and 95% CIs of semen quality parameters associated with apparent temperature exposure during 0–90 days before the date of semen examination for all participants.

Semen quality parameter	Regression coefficients (95% CI)						
	Per 1 °C increase	Quintile of exposure to apparent temperature^a^, °C					*p* for trend^b^
		7.72–11.78	11.79–18.45	18.46–25.61	25.62–31.48	31.49–35.20	
Progressive motility^c^	−2.117 (−2.622, −1.611)	0 (ref)	−3.528 (−8.003, 0.947)	−15.608 (−22.623, −8.593)	−32.051 (−41.614, −22.488)	−45.774 (−56.788, −34.760)	<0.001
Total motility^c^	−4.882 (−6.250, −3.515)	0 (ref)	−6.492 (−18.596, 5.612)	−35.807 (−54.781, −16.832)	−73.980 (−99.848, −48.113)	−107.907 (−137.699, −78.116)	<0.001
Total sperm number^c^	−0.036 (−0.094, 0.021)	0 (ref)	0.033 (−0.475, 0.540)	0.118 (−0.678, 0.913)	0.091 (−0.994, 1.176)	−0.718 (−1.968, 0.531)	0.337
Sperm concentration^c^	−0.013 (−0.055, 0.029)	0 (ref)	−0.059 (−0.433, 0.314)	0.280 (−0.305, 0.865)	0.510 (−0.287, 1.308)	0.048 (−0.871, 0.967)	0.832
Semen volume^c^	−0.007 (−0.014, 0.001)	0 (ref)	0.020 (−0.047, 0.087)	−0.074 (−0.179, 0.031)	−0.164 (−0.307, −0.020)	−0.227 (−0.393, −0.062)	0.010

CIs, confidence intervals. Estimated changes (95% CIs) were estimated using multiple linear regression model, adjusting for age, ever having fathered a child, smoking, alcohol consumption, education, occupation, abstinence periods, season of sperm collection, daily mean precipitation, sunshine duration and air pollutants (PM_2.5_, PM_10_, SO_2_, NO_2_, O_3_, CO) transformed by PCA analysis.

^a^Assessed by averaging daily mean apparent temperatures during 0–90 days before semen examination.

^b^p-Value for linear trend was tested based on variable containing the median value for each quintile.

^c^Box-Cox transformation applied.

**Figure 3:**
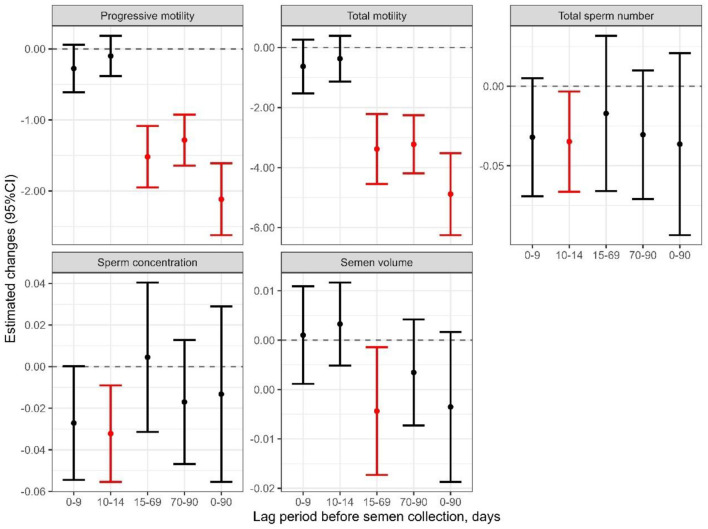
Estimated change and 95% confidence intervals (CIs) of semen parameters associated with each 1 °C increase of lag 0–9, 10–14, 15–69, 70–90 and 0–90 days exposure to apparent temperature for all participants. The vertical gray dashed lines represent a reference level of 0.

Figure 3 despict the estimated alterations in semen quality corresponding to the five exposure periods (0–90, 0–9, 10–14, 15–69, 70–90 days). Sperm motility demonstrated a significant inverse correlation with AT in the 15–69 and 70–90 days lag periods (all *p* < 0.001), with a more pronounced negative association evident in the 0–90 days lag period (Figure 3). In addition, a significant decrease in sperm concentration and total sperm count was exclusively associated with exposure to AT during the lag period of 10–14 days (all *p* < 0.05).

**Figure 4:**
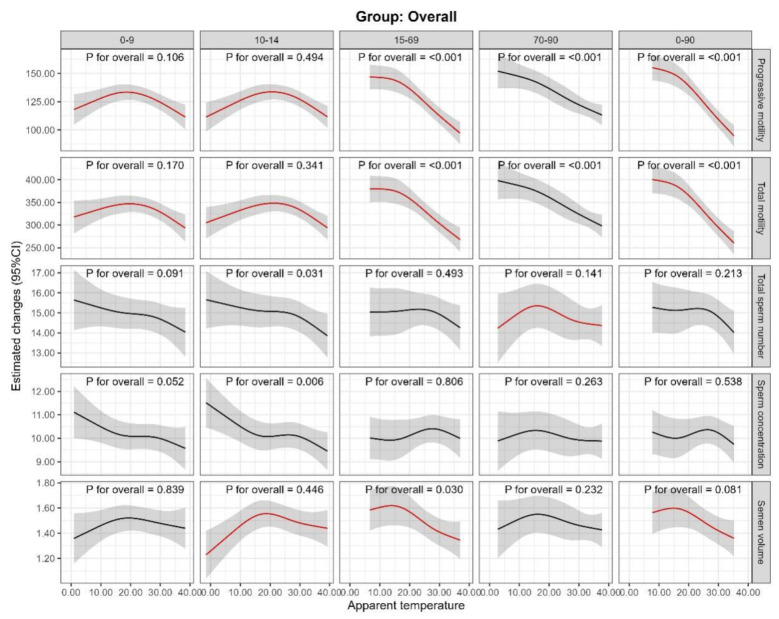
Exposure-response curves of the association between apparent temperature exposure during five periods before semen examination and semen quality parameters for all participants. Data were fitted by a linear regression model using a restricted cubic spline, and the model was conducted with four knots at the 5th, 35th, 65th, 95th percentiles of air pollutants. Red lines indicate *p* for nonlinear <0.05, shadow shape indicate 95% CIs.

The result of the restricted cubic spline analysis depicted an inverted U-shaped non-linear relationship of progressive motility, total motility and AT, especially in lag 0–9 and 10–14 days (Figure 4, all *p* for nonlinear <0.05). The same curvilinear relationship was also observed between AT and semen volume in lag 10–14 days (Figure 4, *p* for nonlinear <0.05). Table 4 presents the outcomes of the segmental analysis conducted using the cut-off values, where the progressive motility, total motility and semen volume were positively associated with AT up to the cutoff value, and negatively associated beyond the cutoff value during lag 10–14 days (all *p* < 0.05), indicating the presence of a threshold effect; therefore, the highest point might represent the optimal AT.

**Table 4:** Estimated changes and 95% CIs of semen quality parameter associated with each 1 °C increased of apparent temperature exposure by cutoff value for all participants.

Semen quality parameter	Cutoff value, °C	Per 1 °C increased^†^	
		≤ Cut-off value	≥Cut-off value
Progressive motility^*^	
0–9 days	18.65	0.4691 (−0.3068, 1.2451)	−1.3507 (−1.9139, −0.7875)
10–14 days	20.66	1.1228 (0.5800, 1.6657)	−1.3532 (−1.9091, −0.7974)
Total motility^*^	
0–9 days	19.53	1.0408 (−0.8661, 2.9476)	−2.4913 (−4.1361, −0.8464)
10–14 days	21.22	2.0548 (0.6548, 3.4548)	−3.2498 (−4.8011, −1.6984)
Semen volume^*^	
10–14 days	18.80	0.0122 (0.0029, 0.0215)	−0.0100 (−0.0170, −0.0030)

CIs, confidence intervals.

Estimated changes (95% CIs) were estimated using multiple linear regression model, adjusting for age, ever having fathered a child, smoking, alcohol consumption, education, occupation, abstinence periods, season of sperm collection, daily mean precipitation, sunshine duration and air pollutants (PM_2.5_, PM_10_, SO_2_, NO_2_, O_3_, CO) transformed by PCA analysis.

^*^Box-Cox transformation applied.

^†^Assessed by averaging daily mean apparent temperatures during 0–9 or 10–14 days before semen examination.

### 3.4 Stratified and sensitivity analyses

Table 5 displays the results of stratification by age and season during 0–90 days before semen examination. We observed that age and season had a significant effect modification on the progressive motility, total motility, sperm concentration and total sperm number (all *p* for interaction <0.001). A larger negative association was identified between AT and progressive motility and total motility in men under the age of 30 and in summer season (all *p* < 0.001). In the stratified analysis by occupation (Table 6), significant main effects were observed only for progressive motility and total sperm motility (both *p* < 0.001). Specifically, non-workers exhibited steeper declines in both progressive motility (β = −2.920, 95%CI: −3.870, −1.970) and total sperm motility (β = −6.951, 95%CI: −9.518, −4.383) compared to workers (β = −1.723 and β = −3.881). Although greater reductions in total sperm count and sperm concentration were also observed in non-workers, and the between-group difference in semen volume was minimal, none of these differences attained statistical significance (all *p* > 0.05). Supplementary Tables S4–S8 and Supplementary Figures S5–S10 present the result of the sensitivity analysis. Across all subgroups, the findings from the exposure-response curves, correlations between exposure and time, and segmented analyses pertaining to sperm motility mirrored those observed in the overall participant population. However, upon conducting the segmentation analysis on these subgroups, it was observed that only the sperm motility during the lag 10–14 days exhibited similarity to that of the overall participant population.

**Table 5:** Estimated change and 95% CIs of semen parameters associated with each 1 °C increase of 0–90 days exposure to apparent temperature stratified by age for all participants.

Semen quality parameter	Age			*p* for interaction	Season				*p* for interaction
	≤ 30	31–39	≥40		Spring	Summer	Autumn	Winter	
Progressive motility	−2.7127 (−3.6238, −1.8017)	−1.8958 (−2.5688, −1.2229)	−2.1917 (−3.6723, −0.7111)	<0.001	0.8172 (−0.6263, 2.2608)	−4.0559 (−4.9609, −3.1509)	−2.1164 (−3.3999, −0.8328)	−1.7137 (−2.6504, −0.7771)	<0.001
Total motility	−6.1998 (−8.6464, −3.7532)	−4.4427 (−6.2709, −2.6144)	−5.0558 (−9.0622, −1.0494)	<0.001	2.0868 (−1.8333, 6.0069)	−9.5311 (−11.9788, −7.0834)	−4.8780 (−8.3481, −1.4079)	−4.1616 (−6.6929, −1.6303)	<0.001
Sperm concentration	−0.0251 (−0.1013, 0.0510)	−0.0183 (−0.0750, 0.0385)	0.0153 (−0.1016, 0.1323)	<0.001	−0.0711 (−0.1891, 0.0469)	−0.0181 (−0.0924, 0.0561)	−0.0223 (−0.1314, 0.0868)	−0.0494 (−0.1300, 0.0311)	<0.001
Total sperm number	−0.0998 (−0.2044, 0.0048)	−0.0374 (−0.1144, 0.0395)	0.0777 (−0.0794, 0.2349)	<0.001	−0.0195 (−0.1819, 0.1429)	−0.1016 (−0.2021, −0.0012)	−0.0300 (−0.1772, 0.1172)	−0.0711 (−0.1814, 0.0391)	<0.001
Semen volume	−0.0230 (−0.0364, −0.0095)	−0.0036 (−0.0139, 0.0067)	0.0159 (−0.0054, 0.0371)	0.363	0.0155 (−0.0062, 0.0373)	−0.0202 (−0.0341, −0.0063)	−0.0019 (−0.0210, 0.0172)	−0.0038 (−0.0183, 0.0107)	0.271

CIs, confidence interval. Estimated changes in semen quality associated with each 1 °C increase in exposure to apparent temperature during 0–90 days before the date of semen examination, stratified by age and season were estimated by multivariate linear models, adjusting for ever having fathered a child, smoking, alcohol consumption, education, occupation, abstinence periods, daily mean precipitation, sunshine duration and air pollutants (PM_2.5_, PM_10_, SO_2_, NO_2_, O_3_, CO) transformed by PCA analysis. Additionally, season (spring, summer, autumn, and winter) was added to the age stratification model, and age (30, 31–39, 40 years) was added to the season stratification model.

**Table 6:** Occupational stratification results of all participants in the 0–90-days apparent temperature exposure window.

Semen quality parameter	Occupation		$p_{interaction}^{*}$
	Workers (*n* = 3,742)	Non-workers (*n* = 1,372)	
Progressive motility	−1.723 (−2.325, −1.120)	−2.920 (−3.870, −1.970)	0.948
Total motility	−3.881 (−5.512, −2.250)	−6.951 (−9.518, −4.383)	0.756
Sperm concentration	−0.020 (−0.089, 0.048)	−0.076 (−0.182, 0.030)	0.091
Total sperm number	0.006 (−0.045, 0.056)	−0.056 (−0.135, 0.023)	0.085
Semen volume	−0.009 (−0.018, 0.000)	−0.003 (−0.017, 0.012)	0.739

Stratified by occupation were estimated by multivariate linear models, adjusting for age, ever having fathered a child, smoking, alcohol consumption, education, abstinence periods, season, daily mean precipitation, sunshine duration and air pollutants (PM_2.5_, PM_10_, SO_2_, NO_2_, O_3_, CO) transformed by PCA analysis. Additionally, occupation (workers, non-workers) was added to the occupation stratification model.

## 4 Discussion

In this retrospective cross-sectional study of 5,114 male individuals from a hospital in Wenzhou during 2014–2022, we investigated both linear and nonlinear exposure–response associations between AT and sperm quality. Our study reveals significant negative linear associations between sperm motility and AT in three exposure periods: 0–90, 15–69, and 70–90 days. An inverse U-shaped relationship was found between sperm motility and AT, particularly within the lag period of 10–14 days. Additionally, we found the strength of the negative association between AT and sperm motility was greatest among individuals aged below 30 years and samples collected in the summer season, and there was no significant difference in semen parameters between the workers' group and the non-workers' group. We also observed comparable findings in the subset of individuals with normal semen quality. Collectively, these findings indicate that while the per-degree change in sperm motility is modest, the cumulative effect of high-temperature exposure may carry clinically relevant implications.

Previous studies have observed a seasonal variation in semen quality, with sperm quality being superior during spring and fall compared to summer, and have also identified ambient temperature as a potential contributing factor (19, 42–46). In recent years, multiple studies have examined the quantitative relationship between ambient temperature and semen quality (15, 17, 18, 28). Zhang et al. (28) reported that elevated ambient temperatures correlated with decreased sperm count, motility, and morphology, with a significant emphasis on the lagged effect of a 10–14-day exposure period on sperm motility decline. The inverted U-shaped curve association observed in current study was also reported by two studies from Wuhan city, although the numerical magnitude of the corresponding threshold effect differed (17, 18). Furthermore, several studies have explored the association between temperature fluctuations and sperm quality. Xiao et al. (15) reported substantial daily temperature changes as inversely correlated with sperm count decreases and Ma et al. (16) uncovered a significantly U-shaped relationship between ambient temperature and the diurnal temperature range in relation to sperm parameters. Deng et al. (47) found a negative relationship between heat waves and sperm quality based on the lag effect of temperature, noting that exposure within the early stage of spermatogenesis (approximately 50 days) was related with a decrease in sperm count and morphology and exposure within the late stage of spermatogenesis (the last 20 days) was related with a decrease in sperm motility.

While the precise biological pathways by which temperature influences semen quality remain incompletely elucidated, it is widely acknowledged that thermal stress can exert detrimental effects on spermatogenesis. The testicular temperature customarily maintains a differential of approximately 4–5 °C lower than the core body temperature (48). Heat stress (HS) occurs when the scrotum is exposed to ambient heat beyond its physiological regulatory limit. It harms sperm cells and diminishes semen quality by triggering the production of reactive oxygen species (ROS), inducing apoptosis, causing DNA damage, and altering gene expression in the testicles (48, 49). In addition, animal studies have shown that exposure to low temperature may also cause cold shock (8), trigger germ cell apoptosis, and result in a reduction in semen quality (9, 50). However, these results still need more evidence to support.

Our findings are consistent with earlier investigations, further supporting a negative association between temperature exposure and semen quality. In terms of clinical relevance, the estimated effect sizes were modest: a 1 °C increase in AT corresponded to an approximate decrease of 0.12 percentage points in progressive motility and 0.08 percentage points in total motility. Although these per-degree changes appear small, several considerations suggest they may carry clinical significance. Temperature exposure is ubiquitous and cumulative, with repeated exposure across daily activities and seasons potentially leading to additive effects. Notably, an inverted U-shaped exposure-response relationship emerged during the late stages of sperm development (lag 0–14 days), suggesting a critical temporal window during which temperature exposure may exert a significant influence. While the underlying mechanisms remain speculative, it is possible that moderate temperature increases within a certain range transiently stimulate compensatory pathways, whereas extreme fluctuations may overwhelm these mechanisms, leading to net negative effects on sperm maturation (28). Stratified analyses revealed that AT had a greater association with semen quality in samples collected during summer, consistent with higher seasonal temperatures, and in younger men (aged <30 years), possibly because younger individuals may be more susceptible to external influences before age-related decline becomes dominant (51, 52). These mechanistic hypotheses, however, require confirmation through experimental studies.

In terms of clinical relevance, the estimated effect sizes were modest: a 1 °C increase in AT corresponded to an approximate decrease of 0.12 percentage points in progressive motility and 0.08 percentage points in total motility. While these per-degree changes appear small, several considerations suggest they may carry clinical significance. First, temperature exposure is ubiquitous and cumulative; individuals are exposed to ambient temperature variations throughout their daily lives and across seasons, leading to repeated and potentially additive effects over time. Second, we identified a critical exposure window of 10–14 days prior to ejaculation, during which even modest temperature increments may interfere with late-stage sperm maturation. Third, the impact was more pronounced in younger men and during summer months, indicating that susceptible subgroups may experience larger cumulative effects. Therefore, although the per-degree change is modest, the combination of ubiquitous exposure, a defined vulnerable window, and population susceptibility suggests that temperature-related effects on sperm quality may be of clinical relevance, particularly for men seeking fertility evaluation. Nonetheless, these findings should be interpreted as associations, and clinical applications would require further validation in prospective studies.

Our findings revealed that although no significant differences in semen parameters were observed between workers and non-workers, the non-worker group exhibited greater potential adverse trends in total sperm count and concentration. This may be attributed to the heterogeneity within the worker group, where variations in job types and exposure levels could mask potential subgroup differences. Additionally, occupational exposure factors are difficult to fully control; workers may be exposed to chemical substances (e.g., heavy metals, organic solvents), noise, or vibration, which could independently affect semen quality (53, 54). Occupational-related behavioral differences may also influence male semen quality. Workers, who often engage in more physical labor and have higher metabolic rates, may develop adaptability to high-temperature environments, whereas non-workers with sedentary occupations (e.g., drivers, office workers) may experience impaired semen quality due to obesity or poor blood circulation (55). Collectively, these findings underscore the need for future investigations employing more precise occupational classification and exposure assessment methodologies.

Notably, AT has a direct and substantial association with public health. Past research has identified AT as potentially the most prominent risk factor for heat-related mortality amidst all causes of death (56), as it is more closely associated with mortality than other temperature variables (57). There is currently ample research indicating that non-optimal AT is associated with negative outcomes in the circulatory and respiratory systems. However, the available evidence regarding the reproductive system, particularly male semen quality, is extremely limited. Our study provided a comprehensive analysis of the linear and nonlinear relationships between AT and semen quality. It presented fresh evidence supporting a negative association between inappropriate temperature exposure on male semen quality, and highlighting the potential importance of minimizing extreme temperature exposure as a precautionary consideration. In addition, based on model-based nonlinear fits, we observed an estimated temperature range of 17.57–22.69 °C associated with higher semen quality in this study population from the urban region of Wenzhou. This relatively narrow and cool range is consistent with certain existing physiological observations. Prior studies indicate that men exhibit a lower thermal discomfort threshold and a reduced tolerance for heat stress compared to women (e.g., a preferred Wet Bulb Globe Temperature of ~22 °C for males vs. ~25 °C for females) (58, 59). This physiological predisposition suggests that the male reproductive system may be particularly vulnerable to temperature elevations, underscoring the practical. Furthermore, men are less likely than women to perceive discomfort related to changes in body temperature (59), when it comes to outdoor thermal exposures, men tend to experience more extreme cold and heat compared to women (60). However, this finding should be interpreted with caution, as it is exploratory in nature and specific to our study population. The observed range may partly reflect the local climate distribution rather than a universal physiological optimum. These findings suggest a potential need for customized strategies to mitigate thermal risk to male reproductive health, although such strategies would require further evaluation. The WHO's housing and health guidelines recommend maintaining indoor temperatures above 18 °C to mitigate the adverse health effects linked to cold indoor conditions (61). Despite this recommendation, numerous households, even in temperate climates, struggle to achieve this standard (62). These findings suggest that in regions with warm and humid climates such as Wenzhou, maintaining thermal conditions conducive to spermatogenesis may present practical challenges. While our observations point to a potential role for indoor temperature regulation in supporting male reproductive health, this hypothesis warrants confirmation through intervention studies or experimental models before any definitive conclusions can be drawn. From a clinical perspective, ambient heat exposure may be considered as one of multiple factors in fertility assessments; however, the current evidence does not yet support specific temperature-based recommendations. Men, especially those seeking to improve fertility, could be advised to prioritize staying within the identified comfortable range (e.g., through the use of air conditioning during heatwaves) and to adopt practical measures such as wearing loose-fitting, breathable clothing to minimize scrotal heat stress during high AT exposure. The range identified in our study provides a preliminary, context-specific reference point that could inform future occupational health guidelines and living environment design considerations, though its applicability to other settings requires further validation.

Several limitations of this study cannot be overlooked when interpreting the findings. First, exposure misclassification is inherent in our study design. The use of city-wide averages as proxies for individual exposure failed to account for participants' mobility patterns, occupational exposure differences, and indoor-outdoor environmental variations. This misclassification is likely non-differential, as it is independent of the outcome (semen quality). Consequently, such non-differential misclassification would bias effect estimates toward the null, making our findings more conservative rather than overestimating the true associations. In addition, the use of lag-period averages may have smoothed out critical short-term temperature fluctuations during key windows of spermatogenesis, thereby reducing statistical power to detect true associations. Additionally, the use of principal component analysis (PCA) to aggregate multiple air pollutants, while reducing dimensionality and addressing multicollinearity, limits the interpretability of individual pollutant effects. Second, although we adjusted for multiple covariates, residual confounding may still exist due to the unavailability of key variables such as body mass index (BMI), occupational heat exposure, physical activity, sauna or hot bath habits, computer use, tight underwear wearing, and chronic diseases. These unmeasured factors are known to influence semen quality and may also be correlated with ambient temperature exposure. As a result, the observed associations may be subject to residual confounding. The direction of this potential bias is difficult to predict, as it depends on the specific relationships between these unmeasured confounders and both exposure and outcome. Depending on the underlying structure, residual confounding could either overestimate or underestimate the true exposure–response associations. Future studies with more comprehensive covariate collection are warranted to address this limitation. Third, the study population was recruited from a reproductive medicine center in Wenzhou, which may introduce referral bias. Men seeking fertility evaluation may differ systematically from the general male population in terms of health status, lifestyle factors, and exposure histories. Such selection mechanisms could bias the observed associations in either direction, depending on how exposure and outcome are distributed in the study population relative to the general population. As a result, the generalizability of our findings to the broader male population, particularly to men without fertility concerns, is limited. Moreover, the exact proportion of participants undergoing fertility evaluation vs. routine health checks could not be precisely ascertained due to the retrospective nature of data collection, further complicating the assessment of sample representativeness. Thus, our results are most directly applicable to men attending a reproductive medicine center in similar climatic and geographic settings. Nevertheless, geographic and population-specific factors should be considered when extrapolating our results to other settings. Future studies with population-based sampling and more representative cohorts are warranted to validate our findings and improve external validity.

## 5 Conclusion

We found a robust association between apparent temperature and compromised semen quality, with the most responsive exposure period corresponding to 10–14 days prior to ejaculation. An inverted U-shaped relationship was observed between apparent temperature and semen quality, with temperatures in the range of 17.57–22.69 °C associated with higher semen quality in this study population.

## Data Availability

The raw data supporting the conclusions of this article will be made available by the authors, without undue reservation.
