# Supplementary material for: Quantifying the exposure-response relationship between temperature exposure and semen quality
Source: Front Public Health. 2026 Apr 13;14:1813888. doi: 10.3389/fpubh.2026.1813888 (PMC13111441; doi:10.3389/fpubh.2026.1813888)
Supplement: Supplementary file 1 [file Table_1.pdf]

Table S1 Demographic characteristics and semen quality of subgroups.

Characteristics	Subgroups		
	Normal semen quality group (N = 3660)	Non- COVID-19 Group (N = 3633)	Delete unknwon value group (N = 4478)
Age, years, n (%)			
≤ 30	1192 (32.6)	1161 (32.0)	1401 (31.3)
31-39	2031 (55.5)	1987 (54.7)	2496 (55.7)
≥ 40	431 (11.8)	474 (13.0)	581 (13.0)
Unknown	6 (0.2)	11 (0.3)	
Ever having fathered a child, n (%)			
Yes	1424 (38.9)	1418 (39.0)	1721 (38.4)
No	2236 (61.1)	2215 (61.0)	2757 (61.6)
Alcohol consumption, n (%)			
Yes	36 (1.0)	32 (0.9)	50 (1.1)
No	3624 (99.0)	3601 (99.1)	4428 (98.9)
Smoking, n (%)			
Yes	247 (6.7)	223 (6.1)	311 (6.9)
No	3413 (93.3)	3410 (93.9)	4167 (93.1)
Occupation, n (%)			
Worker	2657 (72.6)	2604 (71.7)	3246 (72.5)
Businessman	370 (10.1)	331 (9.1)	479 (10.7)
Peasant	235 (6.4)	259 (7.1)	325 (7.3)
Intellectual	27 (0.7)	39 (1.1)	40 (0.9)
Others	292 (8.0)	302 (8.3)	388 (8.7)
Unknown	79 (2.2)	98 (2.7)	
Education, n (%)			
College and higher	1329 (36.3)	1264 (34.8)	1776 (39.7)
High school	517 (14.1)	566 (15.6)	734 (16.4)

Characteristics	Subgroups		
	Normal semen quality group (N = 3660)	Non- COVID-19 Group (N = 3633)	Delete unknwon value group (N = 4478)
Middle school and lower	1415 (38.7)	1424 (39.2)	1968 (43.9)
Unknown	399 (10.9)	379 (10.4)	
Abstinence periods, day, n (%)			
2-3	1169 (31.9)	1154 (31.8)	1433 (32.0)
4-5	1872 (51.1)	1805 (49.7)	2249 (50.2)
6-7	619 (16.9)	674 (18.6)	796 (17.8)
Season, n (%)			
Spring (Mar-May)	1030 (28.1)	973 (26.8)	1251 (27.9)
Summer (Jun-Aug)	987 (27.0)	1026 (28.2)	1225 (27.4)
Autumn (Sep-Nov)	923 (25.2)	949 (26.1)	1113 (24.9)
Winter (Dec-Feb)	720 (19.7)	685 (18.9)	889 (19.9)
Progressive motility, %	61.8 [50.5,72.3]	53.2 [39.2,67.6]	55.6 [41.5,69.1]
Total motility, %	69.4 [59.2,79.7]	61.8 [48.5,75.5]	63.7 [50.4,76.8]
Sperm concentration, $\times 10^6$/ml	86.1 [52.3,135.0]	72.1 [34.9,122.1]	72.2 [35.8,123.6]
Total sperm number, $\times 10^6$	276.8 [165.9,445.6]	216.3 [101.2,376.9]	223.4 [108.9,386.4]
Semen volume, ml	3.3 [2.4,4.3]	3.1 [2.3,4.2]	3.2 [2.3,4.2]

Data were given as number (percent) / median [lower quartile, upper quartile] as indicated.
