# Supplementary material for: Quantifying the exposure-response relationship between temperature exposure and semen quality
Source: Front Public Health. 2026 Apr 13;14:1813888. doi: 10.3389/fpubh.2026.1813888 (PMC13111441; doi:10.3389/fpubh.2026.1813888)
Supplement: Supplementary file 2 [file Table_2.pdf]

Table S2 Summary statistics of meteorological data and air pollutants exposure by lag 0-90, 0-9, 10-14, 15-69 and 70-90 days.

Exposure	Mean ± SD	Minimum	P_{25}	P_{50}	P_{75}	Maximum
Ambient temperature, ℃						
0-90	19.83 ± 6.28	9.43	13.56	20.21	25.93	29.89
0-9	20.14 ± 6.88	4.30	13.98	20.35	26.59	32.11
10-14	20.15 ± 7.05	2.20	14.06	20.58	26.69	32.90
15-69	19.88 ± 6.71	8.49	13.43	20.46	26.18	31.01
70-90	19.48 ± 7.10	5.42	12.90	19.76	26.16	31.64
Apparent temperature, ℃						
0-90	21.77 ± 8.74	7.73	13.05	22.10	30.23	35.20
0-9	22.17 ± 9.61	0.99	13.50	21.61	31.24	38.40
10-14	22.21 ± 9.84	-1.52	13.55	22.10	31.35	39.26
15-69	21.84 ± 9.35	6.65	12.89	22.03	30.87	36.97
70-90	21.32 ± 9.87	2.74	12.03	21.07	30.47	37.75
Precipitation, mm						
0-90	4.89 ± 2.59	0.49	2.69	4.55	6.71	13.30
0-9	5.09 ± 5.74	0.00	1.14	3.43	6.71	37.02
10-14	5.08 ± 7.22	0.00	0.30	2.38	7.16	48.60
15-69	4.85 ± 2.97	0.11	2.59	4.34	6.37	14.29
70-90	4.86 ± 4.18	0.00	2.02	3.61	6.36	27.07
Sunshine duration, h						
0-90	3.52 ± 1.06	1.09	2.78	3.36	4.24	6.70
0-9	3.51 ± 1.93	0.00	1.95	3.35	4.88	9.31
10-14	3.53 ± 2.30	0.00	1.64	3.30	5.24	9.60
15-69	3.55 ± 1.25	1.03	2.71	3.34	4.26	7.48
70-90	3.44 ± 1.58	0.07	2.24	3.24	4.45	7.99
Relative humidity, %						
0-90	76.99 ± 4.72	64.62	73.63	77.74	80.72	85.21

Exposure	Mean ± SD	Minimum	P_{25}	P_{50}	P_{75}	Maximum
0-9	76.99 ± 8.25	49.30	71.80	77.91	83.10	95.80
10-14	77.16 ± 9.36	41.60	71.05	78.40	83.80	98.20
15-69	76.96 ± 5.47	61.00	73.33	77.16	81.11	87.98
70-90	77.02 ± 6.65	52.76	72.86	77.39	81.67	93.63
Wind speed, m/s						
0-90	0.77 ± 0.08	0.51	0.73	0.77	0.83	0.99
0-9	0.77 ± 0.16	0.30	0.65	0.77	0.88	1.37
10-14	0.77 ± 0.20	0.12	0.63	0.77	0.90	1.64
15-69	0.77 ± 0.10	0.47	0.71	0.78	0.84	1.01
70-90	0.77 ± 0.13	0.40	0.68	0.78	0.87	1.08
$PM_{2.5}$, μg/m^3						
0-90	31.79 ± 9.98	15.37	24.32	30.38	37.60	62.23
0-9	31.12 ± 12.45	8.66	21.62	29.03	38.69	99.45
10-14	31.09 ± 13.83	6.67	21.28	28.31	38.30	108.25
15-69	31.71 ± 10.55	14.68	23.74	29.76	38.28	69.23
70-90	32.46 ± 11.68	12.00	23.21	30.74	40.27	80.64
PM_{10}, μg/m^3						
0-90	58.60 ± 14.19	34.11	48.18	56.86	68.89	99.88
0-9	57.81 ± 20.42	15.61	41.60	54.62	70.54	157.73
10-14	57.63 ± 22.85	13.57	41.12	53.48	70.47	166.07
15-69	58.47 ± 15.69	33.05	46.81	55.93	69.54	113.85
70-90	59.54 ± 18.52	24.17	44.61	57.03	71.79	126.33
SO_2, μg/m^3						
0-90	9.28 ± 4.10	4.24	6.09	7.60	12.00	25.28
0-9	9.22 ± 4.82	3.37	5.79	7.46	11.51	36.55
10-14	9.17 ± 5.01	2.77	5.72	7.31	11.23	38.92
15-69	9.24 ± 4.33	4.08	5.94	7.72	11.50	29.40

Exposure	Mean ± SD	Minimum	P_{25}	P_{50}	P_{75}	Maximum
70-90	9.45 ± 4.77	3.95	5.84	7.72	11.61	31.52
NO_2, μg/m^3						
0-90	35.99 ± 8.92	17.66	28.94	35.40	42.27	61.25
0-9	36.01 ± 12.37	5.71	26.50	34.82	44.43	82.68
10-14	35.65 ± 13.28	4.80	25.56	34.59	44.15	86.92
15-69	35.82 ± 10.14	13.82	28.65	34.88	42.36	68.25
70-90	36.51 ± 11.90	6.41	27.94	36.47	45.14	75.18
O_3, μg/m^3						
0-90	111.30 ± 21.96	55.95	92.48	114.57	129.25	152.58
0-9	111.32 ± 31.99	22.09	86.70	111.45	133.98	231.66
10-14	111.67 ± 36.38	17.27	83.37	111.01	136.67	245.57
15-69	111.73 ± 23.96	46.86	93.24	115.44	131.25	159.25
70-90	110.07 ± 28.89	30.87	87.84	110.88	133.35	178.39
CO, mg/m^3						
0-90	0.69 ± 0.16	0.41	0.55	0.67	0.80	1.13
0-9	0.68 ± 0.20	0.35	0.53	0.66	0.80	1.42
10-14	0.68 ± 0.21	0.32	0.53	0.65	0.81	1.57
15-69	0.69 ± 0.17	0.40	0.55	0.66	0.80	1.15
70-90	0.70 ± 0.18	0.38	0.55	0.69	0.81	1.30

SD, standard deviation; P_{25} = the 25th percentile; P_{75} = the 75th percentile.
