# Supplementary material for: Quantifying the exposure-response relationship between temperature exposure and semen quality
Source: Front Public Health. 2026 Apr 13;14:1813888. doi: 10.3389/fpubh.2026.1813888 (PMC13111441; doi:10.3389/fpubh.2026.1813888)
Supplement: Supplementary file 3 [file Table_3.pdf]

Table S3 The results of principal component analysis for six air pollutants ($PM_{2.5}$, PM_{10}, SO_2, NO_2, O_3, CO).

Group	Loadings and importance of components	Comp.1	Comp.2	Comp.3	Comp.4	Comp.5	Comp.6
Overall				**Lag 0-90**			
	$PM_{2.5}$	0.461	0.005	0.171	0.284	0.499	0.655
	PM_{10}	0.452	0.066	0.426	0.257	0.215	-0.705
	NO_2	0.446	0.116	0.375	-0.169	-0.751	0.233
	SO2	0.423	-0.353	-0.203	-0.768	0.240	-0.091
	O_3	-0.185	-0.894	0.349	0.180	-0.100	0.044
	CO	0.414	-0.241	-0.697	0.450	-0.271	-0.096
	Standard deviation	2.122	1.004	0.551	0.296	0.279	0.140
	Proportion of Variance	0.751	0.168	0.051	0.015	0.013	0.003
	Cumulative Proportion	0.751	0.919	0.969	0.984	0.997	1.000
				**Lag 0-9**			
	$PM_{2.5}$	0.473	0.070	0.218	0.237	0.447	0.684
	PM_{10}	0.456	0.128	0.471	0.142	0.246	-0.688
	NO_2	0.450	-0.153	0.290	-0.063	-0.812	0.164
	SO2	0.441	0.120	-0.343	-0.805	0.156	-0.031
	O_3	-0.055	0.968	-0.030	0.082	-0.218	0.062
	CO	0.411	-0.054	-0.727	0.514	-0.095	-0.163
	Standard deviation	2.014	1.021	0.693	0.458	0.412	0.204
	Proportion of Variance	0.676	0.174	0.080	0.035	0.028	0.007
	Cumulative Proportion	0.676	0.850	0.930	0.965	0.993	1.000
				**Lag 10-14**			
	$PM_{2.5}$	0.475	0.079	0.229	0.305	0.407	0.677
	PM_{10}	0.461	0.145	0.461	0.154	0.216	-0.695
	NO_2	0.449	-0.163	0.278	-0.160	-0.801	0.164
	SO2	0.441	0.033	-0.337	-0.783	0.277	-0.013

Group	Loadings and importance of components	Comp.1	Comp.2	Comp.3	Comp.4	Comp.5	Comp.6
	O_3	0.012	0.962	-0.136	0.024	-0.230	0.053
	CO	0.408	-0.141	-0.725	0.493	-0.128	-0.170
	Standard deviation	1.984	1.029	0.695	0.516	0.454	0.220
	Proportion of Variance	0.656	0.176	0.081	0.044	0.034	0.008
	Cumulative Proportion	0.656	0.833	0.913	0.957	0.992	1.000
			**Lag 15-69**				
	$PM_{2.5}$	0.461	0.012	0.149	0.544	0.262	0.633
	PM_{10}	0.452	0.042	0.442	0.300	0.047	-0.711
	NO_2	0.444	0.085	0.393	-0.545	-0.522	0.267
	SO2	0.425	-0.341	-0.237	-0.506	0.621	-0.070
	O_3	-0.185	-0.911	0.307	0.108	-0.161	0.054
	CO	0.413	-0.210	-0.691	0.221	-0.494	-0.119
	Standard deviation	2.101	0.987	0.603	0.351	0.314	0.160
	Proportion of Variance	0.736	0.163	0.061	0.020	0.016	0.004
	Cumulative Proportion	0.736	0.898	0.959	0.979	0.996	1.000
			**Lag 70-90**				
	$PM_{2.5}$	0.466	0.007	0.195	0.544	0.202	0.639
	PM_{10}	0.456	0.011	0.475	0.259	0.027	-0.707
	NO_2	0.447	-0.120	0.312	-0.604	-0.509	0.255
	SO2	0.433	0.272	-0.282	-0.462	0.665	-0.050
	O_3	-0.140	0.944	0.224	0.011	-0.185	0.072
	CO	0.410	0.146	-0.714	0.244	-0.471	-0.141
	Standard deviation	2.046	1.000	0.672	0.426	0.385	0.188
	Proportion of Variance	0.697	0.167	0.075	0.030	0.025	0.006
	Cumulative Proportion	0.697	0.864	0.939	0.969	0.994	1.000
Normal			**Lag 0-90**				
	$PM_{2.5}$	0.462	0.005	0.165	0.139	0.555	0.658

Group	Loadings and importance of components	Comp.1	Comp.2	Comp.3	Comp.4	Comp.5	Comp.6
	PM_{10}	0.452	0.069	0.429	0.199	0.276	-0.700
	NO_2	0.446	0.114	0.379	0.020	-0.768	0.234
	SO2	0.422	-0.359	-0.209	-0.799	0.036	-0.103
	O_3	-0.188	-0.891	0.356	0.199	-0.047	0.047
	CO	0.412	-0.245	-0.689	0.513	-0.152	-0.094
	Standard deviation	2.118	1.002	0.564	0.304	0.285	0.138
	Proportion of Variance	0.748	0.167	0.053	0.015	0.014	0.003
	Cumulative Proportion	0.748	0.915	0.968	0.983	0.997	1.000
			**Lag 0-9**				
	$PM_{2.5}$	0.475	0.060	0.209	0.188	0.465	0.690
	PM_{10}	0.456	0.117	0.478	0.123	0.266	-0.682
	NO_2	0.450	-0.159	0.292	0.006	-0.813	0.162
	SO2	0.440	0.127	-0.346	-0.813	0.080	-0.042
	O_3	-0.047	0.969	-0.015	0.108	-0.208	0.063
	CO	0.410	-0.050	-0.723	0.526	-0.052	-0.166
	Standard deviation	2.009	1.022	0.704	0.459	0.411	0.206
	Proportion of Variance	0.673	0.174	0.082	0.035	0.028	0.007
	Cumulative Proportion	0.673	0.847	0.930	0.965	0.993	1.000
			**Lag 10-14**				
	$PM_{2.5}$	0.476	0.066	0.229	0.254	0.425	0.686
	PM_{10}	0.461	0.131	0.462	0.137	0.247	-0.691
	NO_2	0.449	-0.170	0.282	-0.092	-0.812	0.148
	SO2	0.438	0.039	-0.348	-0.803	0.201	-0.020
	O_3	0.023	0.965	-0.114	0.051	-0.226	0.051
	CO	0.407	-0.133	-0.722	0.511	-0.085	-0.166
	Standard deviation	1.983	1.027	0.701	0.521	0.449	0.219

Group	Loadings and importance of components	Comp.1	Comp.2	Comp.3	Comp.4	Comp.5	Comp.6
	Proportion of Variance	0.656	0.176	0.082	0.045	0.034	0.008
	Cumulative Proportion	0.656	0.831	0.913	0.958	0.992	1.000
			**Lag 15-69**				
	$PM_{2.5}$	0.462	0.016	0.138	0.537	0.278	0.633
	PM_{10}	0.453	0.047	0.442	0.304	0.052	-0.709
	NO_2	0.444	0.082	0.403	-0.538	-0.521	0.271
	SO2	0.424	-0.344	-0.243	-0.515	0.610	-0.079
	O_3	-0.186	-0.909	0.315	0.118	-0.152	0.057
	CO	0.412	-0.214	-0.682	0.226	-0.504	-0.117
	Standard deviation	2.095	0.988	0.616	0.355	0.325	0.159
	Proportion of Variance	0.731	0.163	0.063	0.021	0.018	0.004
	Cumulative Proportion	0.731	0.894	0.957	0.978	0.996	1.000
			**Lag 70-90**				
	$PM_{2.5}$	0.467	0.016	0.190	0.510	0.261	0.646
	PM_{10}	0.454	0.022	0.480	0.261	0.060	-0.701
	NO_2	0.447	-0.113	0.317	-0.549	-0.568	0.250
	SO2	0.430	0.281	-0.294	-0.529	0.605	-0.058
	O_3	-0.152	0.943	0.214	0.034	-0.189	0.074
	CO	0.409	0.137	-0.708	0.298	-0.452	-0.143
	Standard deviation	2.041	0.993	0.687	0.429	0.397	0.190
	Proportion of Variance	0.694	0.164	0.079	0.031	0.026	0.006
	Cumulative Proportion	0.694	0.859	0.937	0.968	0.994	1.000
Non-COVID-19			**Lag 0-90**				
	$PM_{2.5}$	0.455	0.028	0.228	0.259	0.502	0.649
	PM_{10}	0.449	0.043	0.419	0.255	0.199	-0.719
	NO_2	0.448	-0.014	0.325	-0.072	-0.800	0.221

Group	Loadings and importance of components	Comp.1	Comp.2	Comp.3	Comp.4	Comp.5	Comp.6
	SO2	0.422	0.333	-0.217	-0.788	0.194	-0.069
	O_3	-0.205	0.939	0.108	0.229	-0.095	0.047
	CO	0.414	0.063	-0.780	0.434	-0.147	-0.079
	Standard deviation	2.140	0.951	0.558	0.329	0.271	0.157
	Proportion of Variance	0.763	0.151	0.052	0.018	0.012	0.004
	Cumulative Proportion	0.763	0.914	0.966	0.984	0.996	1.000
			**Lag 0-9**				
	$PM_{2.5}$	0.469	0.105	0.225	0.327	0.384	0.681
	PM_{10}	0.455	0.194	0.417	0.239	0.185	-0.700
	NO_2	0.457	-0.092	0.229	-0.115	-0.833	0.149
	SO2	0.442	0.115	-0.220	-0.815	0.280	-0.016
	O_3	-0.077	0.947	-0.213	0.077	-0.206	0.057
	CO	0.402	-0.182	-0.793	0.390	-0.057	-0.143
	Standard deviation	2.019	1.028	0.652	0.483	0.400	0.214
	Proportion of Variance	0.680	0.176	0.071	0.039	0.027	0.008
	Cumulative Proportion	0.680	0.856	0.927	0.966	0.992	1.000
			**Lag 10-14**				
	$PM_{2.5}$	0.471	0.102	0.221	0.400	0.314	0.679
	PM_{10}	0.461	0.181	0.412	0.269	0.135	-0.703
	NO_2	0.457	-0.127	0.195	-0.212	-0.820	0.142
	SO2	0.444	0.040	-0.136	-0.783	0.411	0.001
	O_3	-0.007	0.945	-0.254	-0.015	-0.198	0.046
	CO	0.399	-0.213	-0.813	0.331	-0.050	-0.153
	Standard deviation	1.988	1.039	0.661	0.538	0.435	0.229
	Proportion of Variance	0.659	0.180	0.073	0.048	0.032	0.009
	Cumulative Proportion	0.659	0.839	0.911	0.960	0.991	1.000

Group	Loadings and importance of components	Comp.1	Comp.2	Comp.3	Comp.4	Comp.5	Comp.6
			**Lag 15-69**				
	$PM_{2.5}$	0.454	0.024	0.234	0.437	0.416	0.612
	PM_{10}	0.450	0.076	0.427	0.253	0.079	-0.734
	NO_2	0.447	0.027	0.320	-0.335	-0.715	0.270
	SO2	0.425	0.303	-0.273	-0.670	0.449	-0.050
	O_3	-0.200	0.949	0.046	0.188	-0.137	0.052
	CO	0.414	0.010	-0.764	0.386	-0.298	-0.089
	Standard deviation	2.117	0.950	0.590	0.370	0.316	0.172
	Proportion of Variance	0.747	0.150	0.058	0.023	0.017	0.005
	Cumulative Proportion	0.747	0.898	0.956	0.978	0.995	1.000
			**Lag 70-90**				
	$PM_{2.5}$	0.458	0.049	0.282	0.459	0.326	0.626
	PM_{10}	0.455	0.113	0.431	0.244	0.028	-0.730
	NO_2	0.451	-0.039	0.219	-0.396	-0.728	0.244
	SO2	0.433	0.236	-0.235	-0.649	0.529	-0.029
	O_3	-0.148	0.962	-0.032	0.107	-0.188	0.067
	CO	0.412	-0.040	-0.794	0.374	-0.219	-0.101
	Standard deviation	2.065	0.985	0.616	0.447	0.382	0.195
	Proportion of Variance	0.711	0.162	0.063	0.033	0.024	0.006
	Cumulative Proportion	0.711	0.873	0.936	0.969	0.994	1.000
Delete-unknown			**Lag 0-90**				
	$PM_{2.5}$	0.462	0.005	0.164	0.272	0.511	0.652
	PM_{10}	0.453	0.069	0.427	0.250	0.219	-0.705
	NO_2	0.446	0.118	0.377	-0.148	-0.752	0.240
	SO2	0.424	-0.352	-0.203	-0.775	0.217	-0.093
	O_3	-0.182	-0.894	0.353	0.182	-0.093	0.045

Group	Loadings and importance of components	Comp.1	Comp.2	Comp.3	Comp.4	Comp.5	Comp.6
	CO	0.413	-0.243	-0.695	0.457	-0.262	-0.100
	Standard deviation	2.117	1.006	0.561	0.299	0.284	0.140
	Proportion of Variance	0.747	0.169	0.052	0.015	0.013	0.003
	Cumulative Proportion	0.747	0.916	0.968	0.983	0.997	1.000
			**Lag 0-9**				
	$PM_{2.5}$	0.474	0.068	0.217	0.227	0.452	0.684
	PM_{10}	0.456	0.121	0.472	0.140	0.248	-0.688
	NO_2	0.451	-0.151	0.284	-0.042	-0.815	0.165
	SO2	0.441	0.120	-0.337	-0.811	0.139	-0.033
	O_3	-0.052	0.970	-0.029	0.089	-0.211	0.058
	CO	0.409	-0.054	-0.732	0.512	-0.078	-0.164
	Standard deviation	2.009	1.020	0.702	0.465	0.417	0.205
	Proportion of Variance	0.672	0.173	0.082	0.036	0.029	0.007
	Cumulative Proportion	0.672	0.846	0.928	0.964	0.993	1.000
			**Lag 10-14**				
	$PM_{2.5}$	0.475	0.081	0.224	0.317	0.401	0.675
	PM_{10}	0.461	0.144	0.465	0.163	0.205	-0.694
	NO_2	0.450	-0.163	0.270	-0.171	-0.800	0.169
	SO2	0.441	0.032	-0.325	-0.782	0.296	-0.015
	O_3	0.012	0.962	-0.138	0.015	-0.229	0.051
	CO	0.406	-0.140	-0.732	0.482	-0.130	-0.174
	Standard deviation	1.980	1.029	0.702	0.521	0.457	0.220
	Proportion of Variance	0.653	0.176	0.082	0.045	0.035	0.008
	Cumulative Proportion	0.653	0.830	0.912	0.957	0.992	1.000
			**Lag 15-69**				
	$PM_{2.5}$	0.462	0.014	0.139	0.556	0.243	0.631
	PM_{10}	0.453	0.046	0.442	0.303	0.032	-0.710

Group	Loadings and importance of components	Comp.1	Comp.2	Comp.3	Comp.4	Comp.5	Comp.6
	NO_2	0.444	0.089	0.396	-0.564	-0.495	0.273
	SO2	0.426	-0.339	-0.236	-0.482	0.641	-0.074
	O_3	-0.180	-0.911	0.311	0.103	-0.163	0.056
	CO	0.412	-0.211	-0.690	0.194	-0.507	-0.121
	Standard deviation	2.093	0.992	0.616	0.356	0.320	0.161
	Proportion of Variance	0.730	0.164	0.063	0.021	0.017	0.004
	Cumulative Proportion	0.730	0.894	0.958	0.979	0.996	1.000
				**Lag 70-90**			
	$PM_{2.5}$	0.467	0.012	0.186	0.540	0.208	0.643
	PM_{10}	0.456	0.017	0.474	0.267	0.035	-0.704
	NO_2	0.446	-0.114	0.319	-0.590	-0.524	0.251
	SO2	0.434	0.263	-0.279	-0.479	0.658	-0.050
	O_3	-0.140	0.948	0.208	0.016	-0.182	0.070
	CO	0.410	0.136	-0.720	0.244	-0.464	-0.146
	Standard deviation	2.044	0.997	0.675	0.430	0.392	0.187
	Proportion of Variance	0.696	0.166	0.076	0.031	0.026	0.006
	Cumulative Proportion	0.696	0.862	0.938	0.969	0.994	1.000
