# Supplementary material for: Quantifying the exposure-response relationship between temperature exposure and semen quality
Source: Front Public Health. 2026 Apr 13;14:1813888. doi: 10.3389/fpubh.2026.1813888 (PMC13111441; doi:10.3389/fpubh.2026.1813888)
Supplement: Supplementary file 4 [file Table_4.pdf]

Table S4 Estimated changes and 95% CIs of semen quality parameters associated with apparent temperature exposure during 0–90 days before the date of semen examination for subgroups.

Semen quality parameter	Regression coefficients (95% CI)						
	Per 1 ℃ increase	Quintile of exposure to apparent temperature[a], ℃					*p* for trend[b]
		Q1	Q2	Q3	Q4	Q5	
**Normal group**							
Progressive motility[c]	**-0.4429 (-0.5697, -0.3160)**	Ref.	-0.9409 (-2.0589, 0.1771)	-4.0847 (-5.8268, -2.3425)	-7.9568 (-10.3212, -5.5924)	-9.8388 (-12.5760, -7.1016)	<0.001
Total motility[c]	**-0.8915 (-1.2355, -0.5476)**	Ref.	-1.1865 (-4.2169, 1.8439)	-8.4243 (-13.1466, -3.7020)	-16.8857 (-23.2946, -10.4767)	-20.8882 (-28.3076, -13.4687)	<0.001
Total sperm number[c]	**-0.0085 (-0.0243, 0.0074)**	Ref.	-0.0408 (-0.1806, 0.0991)	-0.1260 (-0.3439, 0.0920)	-0.1430 (-0.4388, 0.1528)	-0.2457 (-0.5881, 0.0967)	0.183
Sperm concentrationr[c]	-0.0042 (-0.0195, 0.0110)	Ref.	-0.0255 (-0.1604, 0.1093)	-0.0699 (-0.2801, 0.1403)	-0.0390 (-0.3243, 0.2463)	-0.0938 (-0.4241, 0.2364)	0.620
Semen volume[c]	-0.0025 (-0.0067, 0.0018)	Ref.	-0.0073 (-0.0449, 0.0302)	-0.0288 (-0.0873, 0.0297)	-0.0581 (-0.1376, 0.0213)	-0.0791 (-0.1711, 0.0128)	0.094
**Non-COVID-19 group**							
Progressive motility[c]	**-10.5936 (-12.9757, -8.2116)**	Ref.	-29.9997 (-51.6466, -8.3529)	-84.5147 (-121.6744, -47.3551)	-143.9497 (-192.0090, -95.8903)	-205.6152 (-259.2137, -152.0168)	<0.001
Total motility[c]	**-48.6262 (-61.7586, -35.4937)**	Ref.	-136.9576 (-256.2175, -17.6976)	-376.6286 (-581.3537, -171.9035)	-657.7801 (-922.5555, -393.0048)	-955.8548 (-1251.1471, -660.5624)	<0.001
Total sperm number[c]	-0.0018 (-0.0172, 0.0137)	Ref.	0.0511 (-0.0889, 0.1911)	0.0669 (-0.1735, 0.3072)	0.1349 (-0.1759, 0.4458)	-0.0514 (-0.3981, 0.2953)	0.834
Sperm concentrationr[c]	0.0061 (-0.0106, 0.0228)	Ref.	0.0286 (-0.1228, 0.1800)	0.1624 (-0.0975, 0.4223)	0.3026 (-0.0335, 0.6387)	0.1512 (-0.2237, 0.5260)	0.424
Semen volume[c]	-0.0068 (-0.0133, -0.0002)	Ref.	0.0212 (-0.0378, 0.0803)	-0.0784 (-0.1797, 0.0230)	-0.1391 (-0.2701, -0.0080)	-0.1840 (-0.3302, -0.0378)	0.020
**Delete unknwon value group**							
Progressive motility[c]	**-21.8888 (-27.1736, -16.6039)**	Ref.	-46.0047 (-92.8550, 0.8455)	-165.1360 (-237.9870, -92.2850)	-335.0459 (-434.4972, -235.5946)	-481.0857 (-595.3986, -366.7728)	<0.001
Total motility[c]	**-43.1124 (-55.2414, -30.9834)**	Ref.	-78.7912 (-186.3310, 28.7487)	-325.3930 (-492.6149, -158.1712)	-663.1655 (-891.4455, -434.8855)	-964.9287 (-1227.3220, -702.5354)	<0.001

Semen quality parameter	Regression coefficients (95% CI)						
	Per 1 ℃ increase	Quintile of exposure to apparent temperature[a], ℃					p for trend[b]
		Q1	Q2	Q3	Q4	Q5	
Total sperm number[c]	-0.0069 (-0.0220, 0.0082)	Ref.	0.0380 (-0.0964, 0.1724)	0.0202 (-0.1888, 0.2291)	0.0659 (-0.2193, 0.3511)	-0.1293 (-0.4572, 0.1985)	0.577
Sperm concentrationr[c]	-0.0018 (-0.0161, 0.0124)	Ref.	0.0155 (-0.1112, 0.1421)	0.0990 (-0.0979, 0.2959)	0.2101 (-0.0587, 0.4789)	0.0671 (-0.2418, 0.3760)	0.561
Semen volume[c]	-0.0043 (-0.0104, 0.0017)	Ref.	0.0221 (-0.0316, 0.0759)	-0.0570 (-0.1406, 0.0266)	-0.1190 (-0.2331, -0.0049)	-0.1514 (-0.2826, -0.0202)	0.032

CIs: confidence intervals; Ref.: reference value.

Estimated changes (95% CIs) were estimated using multiple linear regression model, adjusting for age, ever having fathered a child, smoking, alcohol consumption, education, occupation, abstinence periods, season of sperm collection, daily mean precipitation, sunshine duration and air pollutants ($PM_{2.5}$, PM_{10}, SO_2, NO_2, O_3, CO) transformed by PCA analysis.

Normal group: Q1 (7.73-11.85), Q2 (11.86-18.45), Q3 (18.46-25.56), Q4 (25.57-31.35), Q5 (31.36-35.20);

Non-COVID-19 group: Q1 (7.73-11.93), Q2 (11.94-19.01), Q3 (19.02-26.14), Q4 (26.15-31.58), Q5 (31.59-34.92);

Delete unknwon value group: Q1 (7.73-11.75), Q2 (11.76-18.45), Q3 (18.46-25.58), Q4 (25.59-31.55), Q5 (31.56-35.20).

[a]Assessed by averaging daily mean apparent temperatures during 0–90 days before semen examination.

[b]p-value for linear trend was tested based on variable containing the median value for each quintile.

[c]Box-Cox transformation applied.
