# Supplementary material for: Quantifying the exposure-response relationship between temperature exposure and semen quality
Source: Front Public Health. 2026 Apr 13;14:1813888. doi: 10.3389/fpubh.2026.1813888 (PMC13111441; doi:10.3389/fpubh.2026.1813888)
Supplement: Supplementary file 5 [file Table_5.pdf]

Table S5 Estimated changes and 95% CIs for semen quality parameter associated with each 1 °C increased of apparent temperature exposure by cutoff value.

Semen quality parameter	Cutoff value, ℃	≤ cut-off value	≥ cut-off value
**Normal group**			
Progressive motility*			
0-9 days	19.50	0.0451 (-0.1354, 0.2255)	-0.2414 (-0.3909, -0.0920)
**10-14 days**	**20.34**	**0.2260 (0.0867, 0.3653)**	**-0.2089 (-0.3405, -0.0773)**
Total motility*			
0-9 days	22.69	0.1093 (-0.2755, 0.4941)	-0.6938 (-1.2794, -0.1083)
**10-14 days**	**21.45**	**0.3894 (0.0420, 0.7369)**	**-0.4532 (-0.8386, -0.0677)**
Semen volume*			
10-14 days	17.57	0.0051 (-0.0007, 0.0109)	-0.0053 (-0.0088, -0.0018)
**Non-COVID-19 group**			
Progressive motility*			
0-9 days	18.56	3.0322 (-0.7018, 6.7661)	-8.0887 (-10.8158, -5.3616)
**10-14 days**	**20.77**	**4.4829 (1.8308, 7.1350)**	**-6.6652 (-9.3923, -3.9381)**
Total motility*			
0-9 days	18.91	18.3959 (-0.9819, 37.7738)	-30.6676 (-46.1748, -15.1604)
**10-14 days**	**21.31**	**15.6190 (1.3242, 29.9137)**	**-33.9259 (-49.3003, -18.5515)**
Semen volume*			
10-14 days	19.13	0.0103 (0.0019, 0.0187)	-0.0061 (-0.0125, 0.0003)
**Delete unknwon value group**			
Progressive motility*			
0-9 days	17.72	3.1145 (-4.9145, 11.1434)	-14.8966 (-20.7909, -9.0024)
**10-14 days**	**19.50**	**10.6963 (5.0185, 16.3741)**	**-14.3523 (-20.1355, -8.5690)**
Total motility*			
0-9 days	18.25	5.3864 (-11.5529, 22.3257)	-22.1148 (-36.6677, -7.5620)
**10-14 days**	**19.87**	**16.4041 (3.9729, 28.8354)**	**-28.9996 (-42.6516, -15.3477)**
Semen volume*			
10-14 days	19.87	0.0060 (-0.0013, 0.0134)	-0.0084 (-0.0140, -0.0028)

CI: confidence interval;
Estimated changes (95% CIs) were estimated using multiple linear regression model, adjusting for age, ever having fathered a child, smoking, alcohol consumption, education, occupation, abstinence periods, season of sperm collection, daily mean precipitation, sunshine duration and air pollutants ($PM_{2.5}$, PM_{10}, SO_2, NO_2, O_3, CO) transformed by PCA analysis.
*Box-Cox transformation applied.
