# Supplementary material for: Quantifying the exposure-response relationship between temperature exposure and semen quality
Source: Front Public Health. 2026 Apr 13;14:1813888. doi: 10.3389/fpubh.2026.1813888 (PMC13111441; doi:10.3389/fpubh.2026.1813888)
Supplement: Supplementary file 6 [file Table_6.pdf]

Table S6 Stratified results of age group for the 0–90 days exposure window in the subgroup

Semen quality parameter	Age group			$P_{interaction}$ *
	≤ 30	31-39	≥ 40	
**Normal group**				
Progressive motility	-0.478 (-0.699, -0.258)	-0.463 (-0.636, -0.290)	-0.300 (-0.676, 0.075)	< 0.001
Total motility	-0.931 (-1.530, -0.333)	-0.981 (-1.450, -0.512)	-0.503 (-1.502, 0.496)	< 0.001
Total sperm number	-0.007 (-0.035, 0.022)	-0.013 (-0.035, 0.008)	0.005 (-0.039, 0.049)	0.208
Sperm concentrationr	0.015 (-0.012, 0.043)	-0.014 (-0.034, 0.007)	-0.010 (-0.053, 0.033)	0.078
Semen volume	-0.012 (-0.019, -0.004)	0.000 (-0.005, 0.006)	0.007 (-0.005, 0.020)	0.929
**Non-COVID-19 group**				
Progressive motility	-13.780 (-18.095, -9.465)	-9.184 (-12.368, -6.001)	-11.285 (-18.201, -4.369)	< 0.001
Total motility	-63.974 (-87.561, -40.387)	-42.652 (-60.298, -25.005)	-49.422 (-87.487, -11.357)	< 0.001
Total sperm number	-0.020 (-0.049, 0.008)	-0.002 (-0.022, 0.019)	0.038 (-0.005, 0.081)	0.488
Sperm concentrationr	-0.004 (-0.035, 0.026)	0.005 (-0.017, 0.028)	0.032 (-0.014, 0.078)	0.329
Semen volume	-0.018 (-0.029, -0.006)	-0.006 (-0.015, 0.003)	0.014 (-0.005, 0.033)	0.500
**Delete unknwon value grou**				
Progressive motility	-27.843 (-37.577, -18.110)	-19.590 (-26.584, -12.596)	-22.390 (-37.463, -7.317)	< 0.001
Total motility	-55.912 (-78.042, -33.782)	-38.579 (-54.691, -22.467)	-43.190 (-78.056, -8.325)	< 0.001
Total sperm number	-0.021 (-0.049, 0.007)	-0.011 (-0.031, 0.010)	0.028 (-0.014, 0.070)	0.548
Sperm concentrationr	-0.008 (-0.035, 0.018)	-0.005 (-0.024, 0.014)	0.013 (-0.026, 0.052)	0.490
Semen volume	-0.013 (-0.024, -0.002)	-0.004 (-0.013, 0.004)	0.017 (-0.001, 0.035)	0.393

* $P_{interaction}$: The P value of the interaction effect was obtained by including the product term of age and apparent temperature in the multiple linear regression model. All semen quality parameters were transformed using the Box-Cox method.。
