# Supplementary material for: Quantifying the exposure-response relationship between temperature exposure and semen quality
Source: Front Public Health. 2026 Apr 13;14:1813888. doi: 10.3389/fpubh.2026.1813888 (PMC13111441; doi:10.3389/fpubh.2026.1813888)
Supplement: Supplementary file 7 [file Table_7.pdf]

Table S7 Stratified results of semen collection seasons for the 0–90 days exposure window in the subgroup

Semen quality parameters	Season				$P_{interaction}$*
	Spring	Summer	Autumn	Winter	
**Normal group**					
Progressive motility	0.162 (-0.203, 0.526)	-0.741 (-0.963, -0.518)	-0.464 (-0.774, -0.155)	-0.440 (-0.685, -0.194)	< 0.001
Total motility	0.299 (-0.688, 1.286)	-1.527 (-2.135, -0.920)	-0.970 (-1.809, -0.131)	-0.913 (-1.579, -0.247)	< 0.001
Total sperm number	-0.027 (-0.072, 0.017)	0.000 (-0.027, 0.028)	-0.001 (-0.041, 0.039)	-0.028 (-0.059, 0.002)	0.242
Sperm concentrationr	-0.047 (-0.089, -0.004)	0.012 (-0.015, 0.039)	-0.005 (-0.044, 0.034)	-0.019 (-0.049, 0.011)	0.164
Semen volume	0.009 (-0.003, 0.022)	-0.007 (-0.014, 0.001)	0.002 (-0.008, 0.012)	-0.004 (-0.012, 0.004)	0.989
**Non-COVID-19 group**					
Progressive motility	1.234 (-5.351, 7.819)	-19.670 (-23.989, -15.351)	-10.467 (-16.091, -4.842)	-8.678 (-13.362, -3.994)	< 0.001
Total motility	6.196 (-30.200, 42.591)	-90.381 (-114.227, -66.534)	-48.726 (-79.908, -17.544)	-42.500 (-68.387, -16.612)	< 0.001
Total sperm number	-0.013 (-0.054, 0.028)	-0.026 (-0.054, 0.002)	0.006 (-0.031, 0.043)	0.003 (-0.030, 0.037)	0.073
Sperm concentrationr	-0.028 (-0.073, 0.017)	-0.006 (-0.036, 0.024)	0.012 (-0.029, 0.052)	0.007 (-0.028, 0.043)	0.361
Semen volume	0.011 (-0.006, 0.029)	-0.019 (-0.031, -0.007)	-0.004 (-0.019, 0.011)	-0.003 (-0.017, 0.011)	0.676
**Delete unknwon value grou**					
Progressive motility	7.622 (-7.436, 22.681)	-40.515 (-49.981, -31.048)	-25.454 (-38.495, -12.413)	-18.653 (-28.504, -8.803)	< 0.001
Total motility	17.185 (-17.498, 51.867)	-80.761 (-102.392, -59.130)	-50.695 (-80.621, -20.768)	-38.793 (-61.523, -16.063)	< 0.001
Total sperm number	-0.005 (-0.046, 0.036)	-0.024 (-0.051, 0.004)	0.002 (-0.036, 0.040)	-0.013 (-0.042, 0.016)	0.140
Sperm concentrationr	-0.019 (-0.057, 0.019)	-0.006 (-0.031, 0.020)	0.003 (-0.034, 0.039)	-0.010 (-0.038, 0.017)	0.516
Semen volume	0.013 (-0.004, 0.030)	-0.016 (-0.027, -0.005)	0.001 (-0.014, 0.015)	-0.002 (-0.014, 0.010)	0.735

* $P_{interaction}$: The P value of the interaction effect was obtained by including the product term of season and apparent temperature in the multiple linear regression model. All semen quality parameters were transformed using the Box-Cox method.
