# Supplementary material for: Quantifying the exposure-response relationship between temperature exposure and semen quality
Source: Front Public Health. 2026 Apr 13;14:1813888. doi: 10.3389/fpubh.2026.1813888 (PMC13111441; doi:10.3389/fpubh.2026.1813888)
Supplement: Supplementary file 8 [file Table_8.pdf]

Table S8 Stratified results of occupation for the 0–90 days exposure window in the subgroup

Semen quality parameters	Occupation		$P_{interaction}$*
	Workers	Non-workers	
**Normal group**	*n* = 2657	*n* = 1003	
Progressive motility	-0.425 (-0.577, -0.274)	-0.507 (-0.744, -0.270)	0.388
Total motility	-0.854 (-1.264, -0.445)	-1.025 (-1.672, -0.377)	0.525
Total sperm number	-0.007 (-0.026, 0.012)	-0.013 (-0.042, 0.016)	0.094
Sperm concentrationr	0.001 (-0.017, 0.020)	-0.017 (-0.046, 0.011)	0.103
Semen volume	-0.004 (-0.009, 0.001)	0.001 (-0.007, 0.009)	0.590
**Non-COVID-19 group**	*n* = 2604	*n* = 1029	
Progressive motility	-9.188 (-12.079, -6.298)	-13.235 (-17.550, -8.921)	0.409
Total motility	-40.746 (-56.664, -24.827)	-64.137 (-87.994, -40.281)	0.672
Total sperm number	0.004 (-0.015, 0.023)	-0.015 (-0.042, 0.012)	0.361
Sperm concentrationr	0.013 (-0.008, 0.033)	-0.009 (-0.039, 0.020)	0.240
Semen volume	-0.008 (-0.016, 0.000)	-0.005 (-0.017, 0.007)	0.862
**Delete unknwon value grou**	*n* = 3246	*n* = 1232	
Progressive motility	-17.886 (-24.165, -11.608)	-30.305 (-40.256, -20.355)	0.680
Total motility	-34.231 (-48.677, -19.785)	-61.579 (-84.269, -38.889)	0.526
Total sperm number	-0.003 (-0.021, 0.015)	-0.012 (-0.041, 0.016)	0.381
Sperm concentrationr	0.003 (-0.014, 0.020)	-0.010 (-0.036, 0.017)	0.237
Semen volume	-0.006 (-0.013, 0.001)	0.000 (-0.012, 0.011)	0.863

* $P_{interaction}$: The P value of the interaction effect was obtained by including the product term of occupation and apparent temperature in the multiple linear regression model. All semen quality parameters were transformed using the Box-Cox method.
