# Supplementary material for: Quantifying the exposure-response relationship between temperature exposure and semen quality
Source: Front Public Health. 2026 Apr 13;14:1813888. doi: 10.3389/fpubh.2026.1813888 (PMC13111441; doi:10.3389/fpubh.2026.1813888)
Supplement: Supplementary file 9 [file Table_9.pdf]

Supplement 1: Box-Cox transformation

The general form of Box-Cox transformation is as follows:

$$y^{(\lambda)} = \begin{cases} \frac{(y+a)^{\lambda}-1}{\lambda}, \lambda \neq 0 \\ In(y+a), \lambda = 0 \end{cases}$$

Where y is the original continuous dependent variable, $y^{(\lambda)}$ is the new variable obtained after the Box–Cox transformation, and λ is the transformation parameter. The above transformation requires that the variable y take on a positive value. If the value of the formula (y+a) positive, and then the above transform is performed.

In this study, we applied the Box–Cox transformation to several semen quality parameters, including sperm concentration, total sperm number, progressive motility, total motility, and semen volume. The optimal transformation parameter λ for each parameter was determined using the 'BoxCox.lambda' function from the forecast package in R. Subsequently, each parameter was transformed accordingly. The distributions of these parameters before and after transformation are illustrated in Supplementary Figures 1.1–S1.4, demonstrating the effectiveness of the transformation in improving normality and stabilizing variance.

Supplementary Table S1 Box-Cox transformation parameters (λ) and transformed means and standard deviations for semen quality parameters across study subgroups

Variable	Dataset	N	Lambda	Mean_transformed	SD_transformed
Progressive motility	Overall	5114	1.725	599.843	333.515
Semen volume	Overall	5114	0.185	1.253	0.560
Sperm concentration	Overall	5114	0.070	4.789	1.347
Total motility	Overall	5114	2.000	2035.120	1119.991
Total spermnumber	Overall	5114	0.065	6.245	1.494
Progressive motility	Normal	3660	0.902	44.298	9.129
Semen volume	Normal	3660	-0.607	0.822	0.186
Sperm concentration	Normal	3660	-0.154	3.182	0.354
Total motility	Normal	3660	1.585	527.655	152.598
Total spermnumber	Normal	3660	-0.157	3.706	0.300
Progressive motility	Non-COVID19	3633	1.604	374.422	206.375
Semen volume	Non-COVID19	3633	0.127	1.188	0.544
Sperm concentration	Non-COVID19	3633	0.071	4.765	1.373
Total motility	Non-COVID19	3633	2.000	1964.647	1111.896
Total spermnumber	Non-COVID19	3633	0.028	5.568	1.279
Progressive motility	Del-unknown	4478	1.825	867.546	495.192
Semen volume	Del-unknown	4478	0.180	1.254	0.555
Sperm concentration	Del-unknown	4478	0.059	4.665	1.289
Total motility	Del-unknown	4478	2.000	2053.042	1116.625
Total spermnumber	Del-unknown	4478	0.049	5.965	1.379
