# Supplementary material for: Quantifying the exposure-response relationship between temperature exposure and semen quality
Source: Front Public Health. 2026 Apr 13;14:1813888. doi: 10.3389/fpubh.2026.1813888 (PMC13111441; doi:10.3389/fpubh.2026.1813888)
Supplement: Supplementary file 13 [file Image_4.pdf]

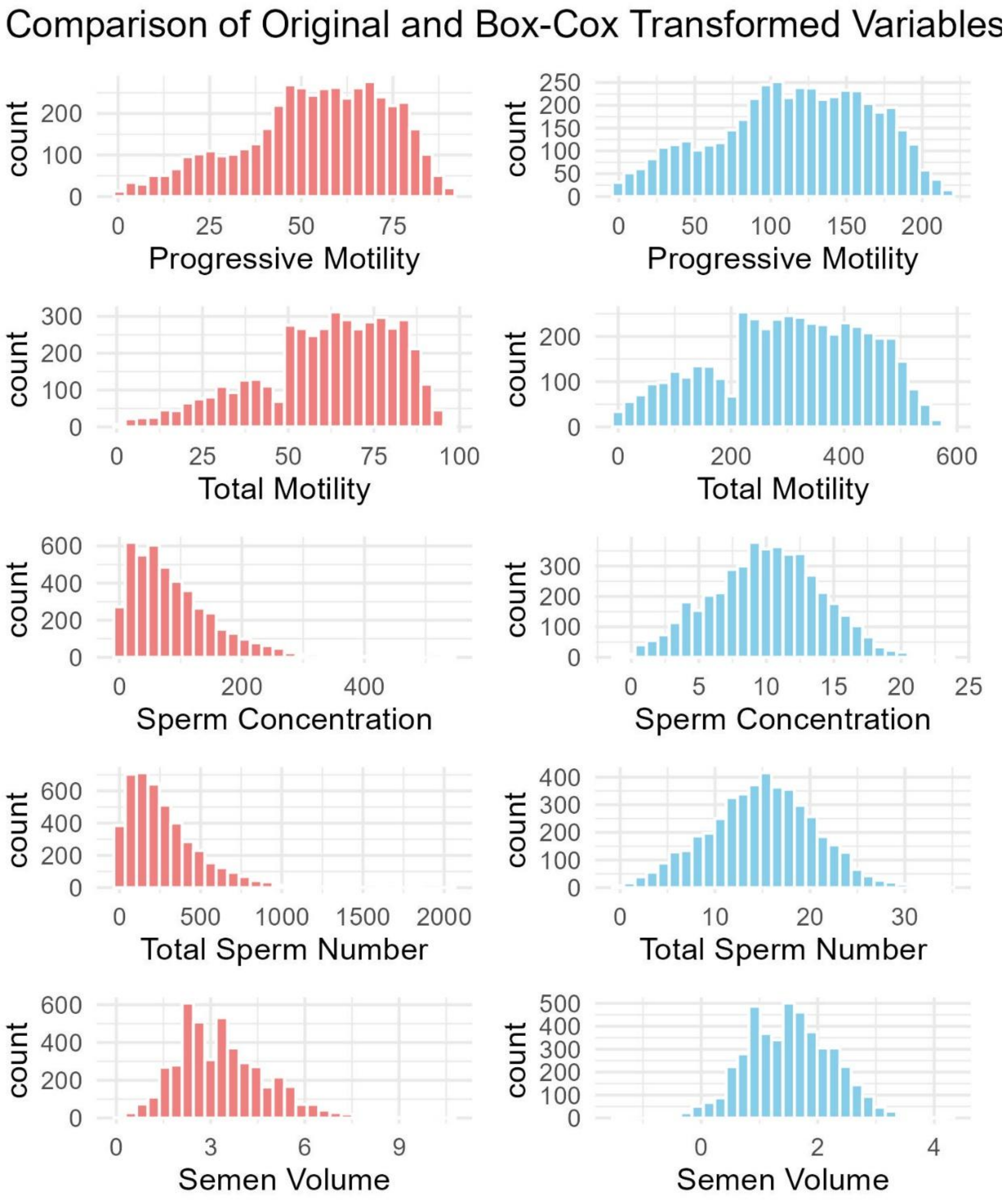

Supplement Figure 1.4 Comparison of the distributions of semen quality parameters for *del-known group* before and after Box-Cox transformation. Red histograms represent the original data, while blue histograms represent the transformed data.
