# Supplementary figures and images for: Quantifying the exposure-response relationship between temperature exposure and semen quality
Source: Front Public Health. 2026 Apr 13;14:1813888. doi: 10.3389/fpubh.2026.1813888 (PMC13111441; doi:10.3389/fpubh.2026.1813888)

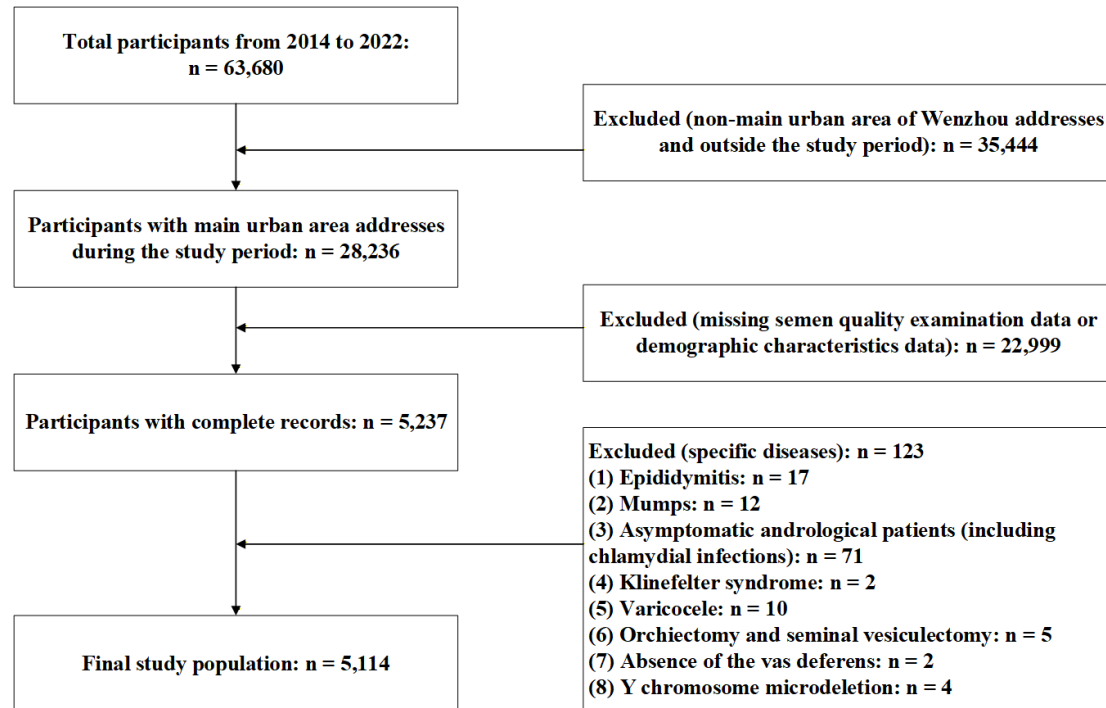

**Fig. S1.** Flowchart of selected 5114 males from the cross-sectional study between 2014-2022 in Wenzhou, China.

Supplement: Supplementary file 14 [file Image_5.pdf]

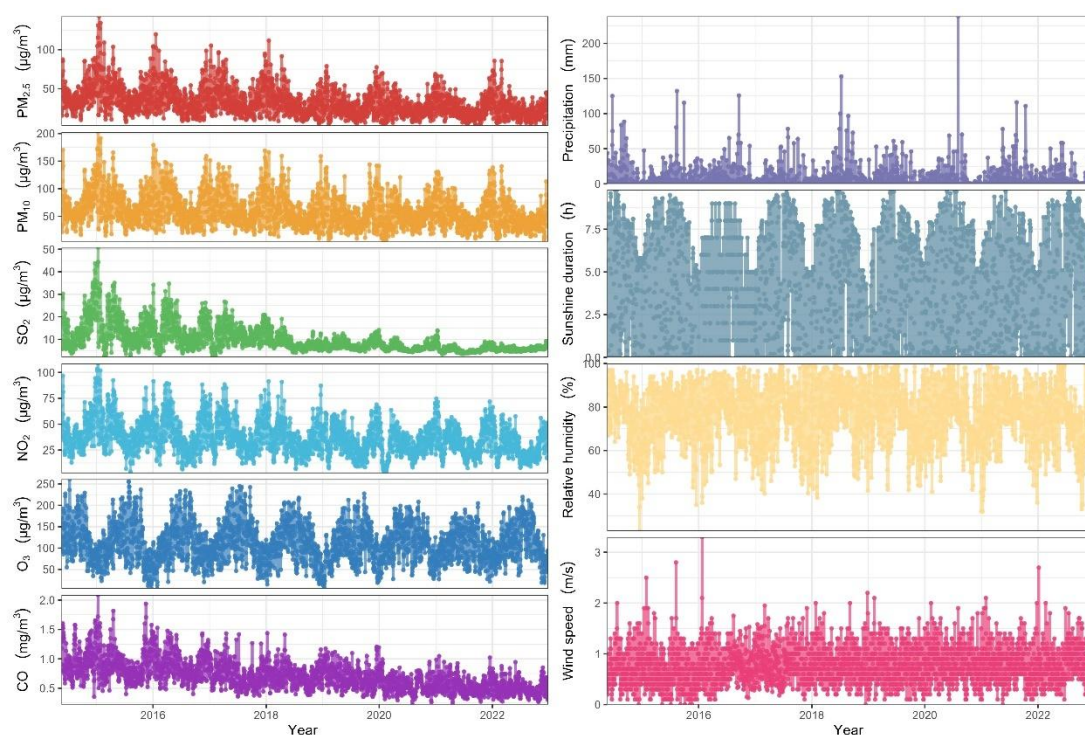

**Fig. S4.** Distributions of air pollutants and meteorological data between 2014 to 2022.

Supplement: Supplementary file 17 [file Image_8.pdf]
