# Supplementary material for: Quantifying the exposure-response relationship between temperature exposure and semen quality
Source: Front Public Health. 2026 Apr 13;14:1813888. doi: 10.3389/fpubh.2026.1813888 (PMC13111441; doi:10.3389/fpubh.2026.1813888)
Supplement: Supplementary file 15 [file Image_6.pdf]

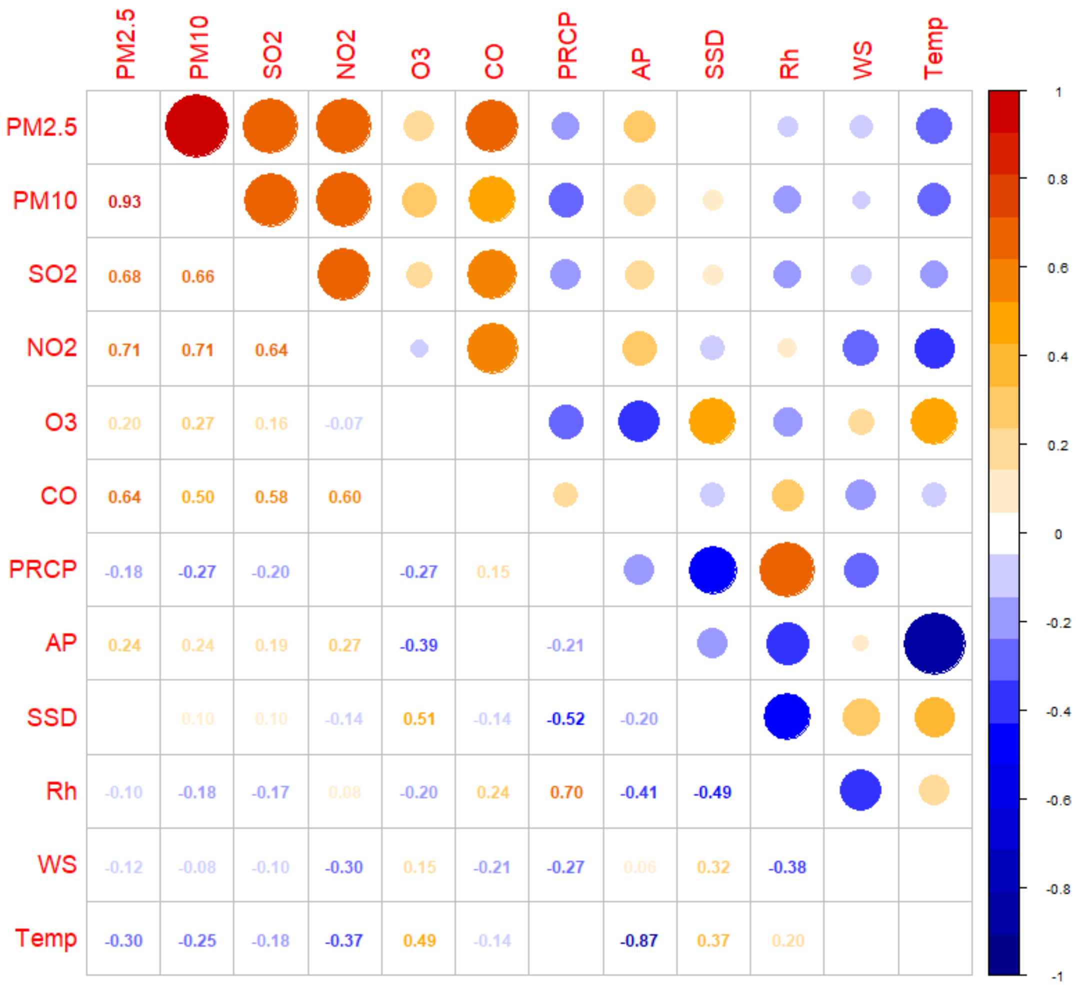

Fig.S3. Spearman correlation between air pollutants and meteorological data. PRCP: precipitation (mm), AP: atmospheric pressure (hPa), SSD: sunshine duration (h), Rh: relative humidity (%), WS: wind speed (m/s), Temp: ambient temperature (°C).
