# Supplementary material for: Quantifying the exposure-response relationship between temperature exposure and semen quality
Source: Front Public Health. 2026 Apr 13;14:1813888. doi: 10.3389/fpubh.2026.1813888 (PMC13111441; doi:10.3389/fpubh.2026.1813888)
Supplement: Supplementary file 16 [file Image_7.pdf]

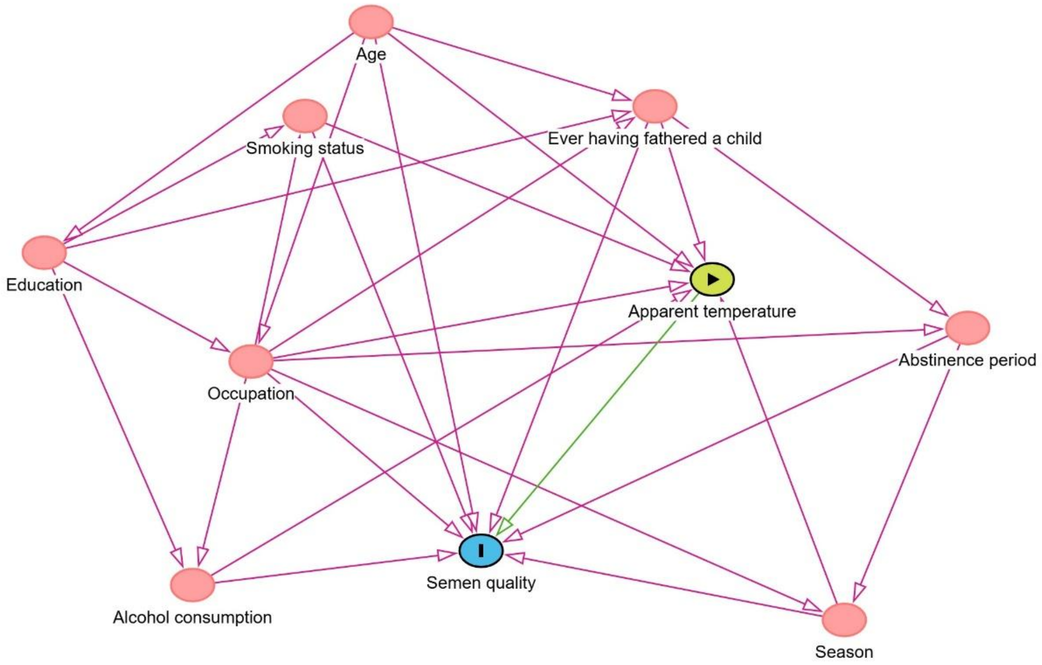

Fig. S2. Directed acyclic graph for evaluation of covariates selection in the association between apparent temperature and semen quality.
