# Supplementary material for: Quantifying the exposure-response relationship between temperature exposure and semen quality
Source: Front Public Health. 2026 Apr 13;14:1813888. doi: 10.3389/fpubh.2026.1813888 (PMC13111441; doi:10.3389/fpubh.2026.1813888)
Supplement: Supplementary file 20 [file Image_11.pdf]

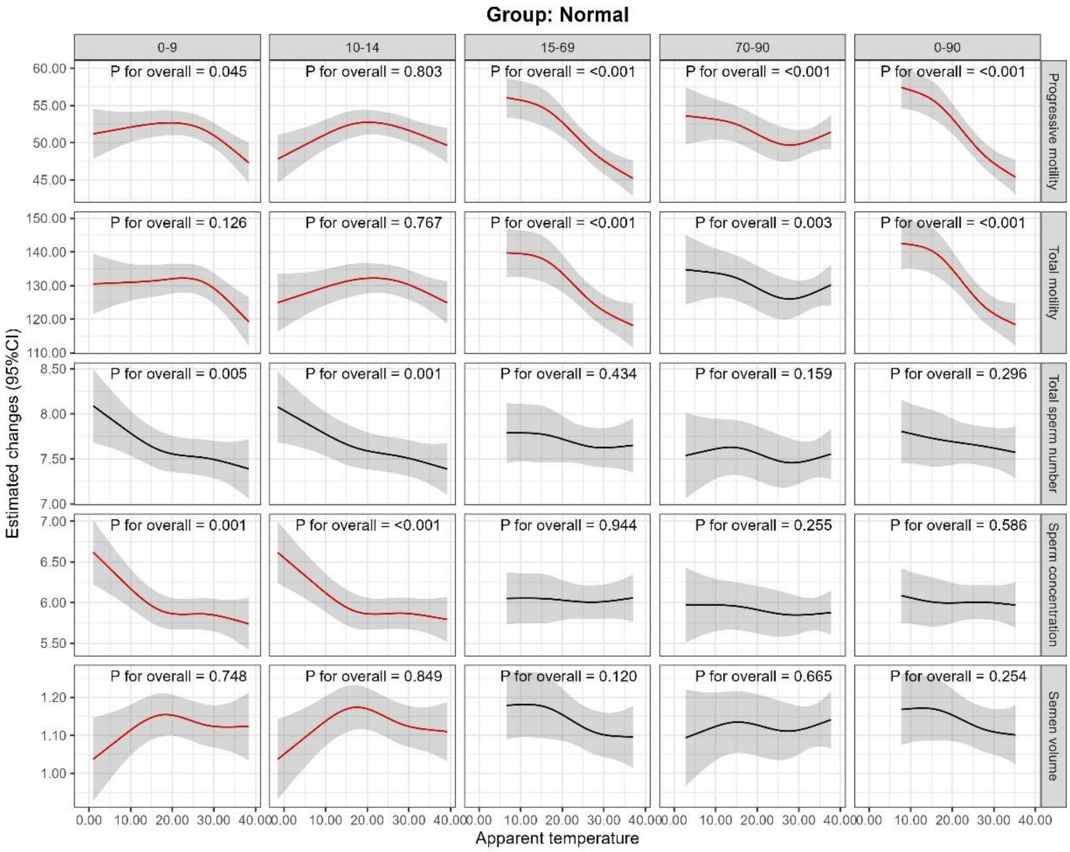

Fig. S7. Exposure-response curves of the association between apparent temperature exposure during five periods before semen examination and semen quality parameters for the *normal semen quality group*. Data were fitted by a linear regression model using a restricted cubic spline, and the model was conducted with 4 knots at the 5th, 35th, 65th, 95th percentiles of air pollutants. Red lines indicate p for nonlinear < 0.05, shadow shape indicate 95% CIs.
